# The pleiotropic spectrum of proximal 16p11.2 CNVs

**DOI:** 10.1016/j.ajhg.2024.08.015

**Published:** 2024-09-26

**Authors:** Chiara Auwerx, Zoltán Kutalik, Alexandre Reymond

**Affiliations:** 1 Center for Integrative Genomics, University of Lausanne, Lausanne, Switzerland; 2 Department of Computational Biology, University of Lausanne, Lausanne, Switzerland; 3 Swiss Institute of Bioinformatics, Lausanne, Switzerland; 4 University Center for Primary Care and Public Health, Lausanne, Switzerland

**Keywords:** proximal 16p11.2 BP4-5 CNV, structural variant, pleiotropy, multi-system disorder, penetrance, variable expressivity

## Abstract

Recurrent genomic rearrangements at 16p11.2 BP4-5 represent one of the most common causes of genomic disorders. Originally associated with increased risk for autism spectrum disorder, schizophrenia, and intellectual disability, as well as adiposity and head circumference, these CNVs have since been associated with a plethora of phenotypic alterations, albeit with high variability in expressivity and incomplete penetrance. Here, we comprehensively review the pleiotropy associated with 16p11.2 BP4-5 rearrangements to shine light on its full phenotypic spectrum. Illustrating this phenotypic heterogeneity, we expose many parallels between findings gathered from clinical versus population-based cohorts, which often point to the same physiological systems, and emphasize the role of the CNV beyond neuropsychiatric and anthropometric traits. Revealing the complex and variable clinical manifestations of this CNV is crucial for accurate diagnosis and personalized treatment strategies for carrier individuals. Furthermore, we discuss areas of research that will be key to identifying factors contributing to phenotypic heterogeneity and gaining mechanistic insights into the molecular pathways underlying observed associations, while demonstrating how diversity in affected individuals, cohorts, experimental models, and analytical approaches can catalyze discoveries.

## Graphical abstract

**Figure:**
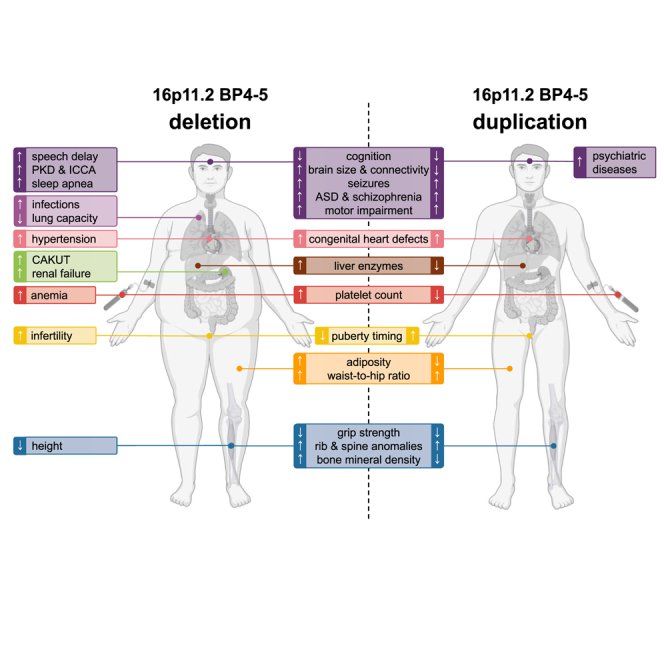

## Teaser

16p11.2 BP4-5 copy-number variants lead to variable clinical presentation, ranging from neuropsychiatric to metabolic disorders. We review data from clinical and population cohorts to shed light on the spectrum of phenotypic alterations observed among carrier individuals and discuss areas of research that will be key to understanding phenotypic heterogeneity.

## Hallmarks of 16p11.2 BP4-5 rearrangements

**Figure 1:**
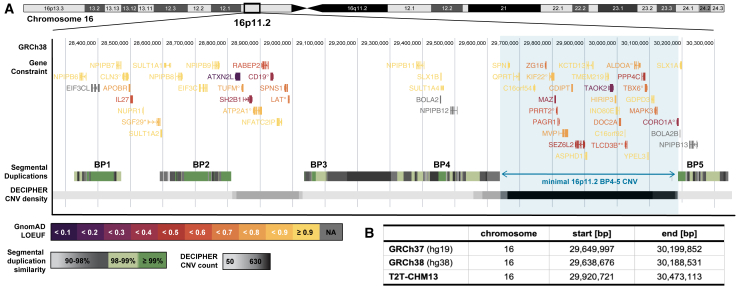
Genomic landscape of the 16p11.2 region (A) Overview of 16p11.2 cytoband (GRCh38), with the minimal 16p11.2 BP4-5 region highlighted in blue. Upper track: exonic structure of protein-coding genes overlapping the region colored according to GnomAD v.2.1.1 loss-of-function observed over expected upper bound fraction (LOEUF) score. Small LOEUF (<0.35) indicates selection against loss-of-function variants in the gene, i.e., evolutionary constraint. Genes with no LOEUF score are in gray. Tagged genes: ° indicates Online Mendelian Inheritance in Man (OMIM) morbid genes; ^∗^ have a new HGNC symbol since the GnomAD v.2.1.1 release (*SGF29* [MIM: 613374] was *CCDC101* and *TLCD3B* [MIM: 615175] was *FAM57B*). Middle track: segmental duplications colored according to similarity degree, ranging from 90% to ≥99%. These form the breakpoints (BP) for recurrent copy-number variants (CNVs). Lower track: density of CNVs reported in the Database of Chromosomal Imbalance and Phenotype in Humans Using Ensembl Resources (DECIPHER; accessed December 12^th^, 2020) colored according to CNV count. While rearrangements of the BP4-5 interval are the most common, rearrangements between other BPs have been described, e.g., the second most common CNV in the region spans a 220 kb interval between the BP2-3 (MIM: 613444). (B) ClinGen coordinates for the minimal region affected by the 16p11.2 BP4-5 rearrangements in three human reference genome builds. Coordinates in GRCh37 were lifted over with the University of California Santa Cruz (UCSC) LiftOver tool. Because breakpoints might occur at several locations within the segmental duplication region, exact coordinates and length might vary across individuals.

**Figure 2:**
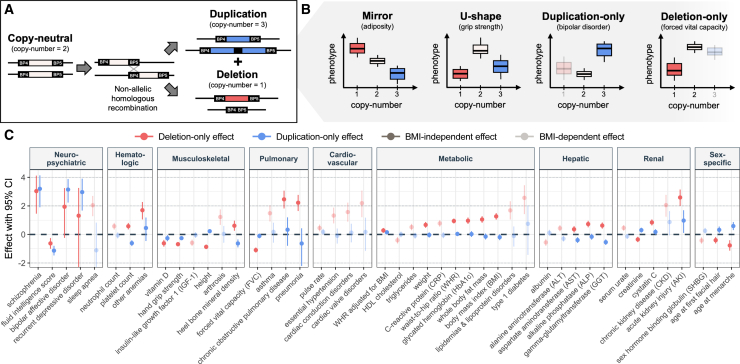
Pleiotropy of the 16p11.2 BP4-5 region in the UK Biobank (A) Most common copy-number states for the 16p11.2 BP5-4 locus, including the copy-neutral state (2 copies; white), deletion (1 copy; red), and duplication (3 copies; blue), which typically arise through non-allelic homologous recombination (NAHR). (B) Schematic representation of the phenotypic distribution, shown as boxplots, of individuals with different 16p11.2 BP5-4 copy-number states according to four dosage mechanisms, with one example phenotype: an additive mirror mechanism wherein deletion and duplication affect the phenotype in opposite direction, a U-shape mechanism wherein any deviation from the copy-neutral state affects the phenotype in the same direction, and a duplication-only or deletion-only mechanism wherein only duplication or deletion carrier individuals deviate from the copy-neutral phenotypic distribution, respectively. For the two last models, deletion and duplication carrier individuals (semi-transparent) are not assessed to obtain the effect of the duplication and deletion, respectively. (C) Effect sizes (beta; y axis) with 95% confidence interval (CI) of the 16p11.2 BP4-5 deletion (red) and duplication (blue) on 46 complex traits and diseases that were significantly (*p* ≤ 0.05/117 = 4.3 × 10^−4^) associated with the region’s copy-number in the UK Biobank through at least one of four tested association models in (B), ordered by physiological system (x axis). Data from Auwerx et al.^28^ Effect sizes are in standard deviation units of the outcome (quantitative traits) or logarithms of the odds ratio of a logistic regression (disease traits). Associations that fail to reach the significance threshold upon conditioning on body mass index (BMI) or involve traits highly correlated with BMI (>0.7) are semi-transparent (BMI-dependent) while the others are opaque (BMI-independent).

Chromosome 16 is particularly rich in segmental duplications, which are typically defined as clusters of repeated sequences larger than 1 kb.^1^^,^^2^ Due to their high sequence similarity (≥90%), segmental duplications are prone to misalignment during meiosis, promoting *de novo* CNV formation through non-allelic homologous recombination (NAHR). As such, segmental duplications cradle the breakpoints (BPs) of recurrent genomic rearrangements. These rearrangements are at the origin of genomic disorders through the deletion and reciprocal duplication of one or more **dosage-sensitive** genes^3^ (Box 1). The 16p11.2 **cytoband** (Box 1) comprises five segmental duplication clusters termed BP1-5 (Figure 1A), two of which (BP4 and BP5) underwent a rapid, *Homo sapiens*-specific expansion that favors the creation of proximal 16p11.2 copy-number variations (CNVs [MIM: 611913, 614671]).^4^ Exact breakpoints vary between individuals, but the recurrent 16p11.2 BP4-5 CNV encompasses a core region of ∼600 kb, which overlaps 27 unique protein-coding genes, as well as 4 multi-copy genes mapping to the repetitive flanking regions (Figures 1A and 1B). In contrast to some other genomic disorders, expression of 16p11.2 BP4-5 genes is positively correlated with the region’s dosage,^5^^,^^6^ with no dosage compensation. Hinting at the deleterious potential of these CNVs, some of the encompassed genes are under **evolutionary constraint** (Box 1) and/or have been linked to Mendelian disorders^7^ (Figure 1A). Accordingly, multiple mouse models ablated for single 16p11.2 BP4-5 orthologs show embryonic or preweaning lethality (Box 2; Table S1). While no homozygous 16p11.2 BP4-5 deletion has been reported, suggesting lethality, triplication—either in tandem^8^ or due to biparental inheritance^9^^,^^10^—has been reported in four individuals. More common is the loss or gain of a single copy, resulting in a heterozygous deletion and duplication (Figure 2A). These will be the focus of this review.

Box 1 **Genetic glossary**

**Ascertainment bias:** Sampling bias leading some individuals to be more or less likely to be included in a study or cohort, so that the resulting sample is not fully representative of the targeted population.

**Burden test:** Joint analysis of multiple rare variants meeting certain criteria that are grouped into a single analysis unit, typically a gene, to perform an association study with a selected phenotype. The optimal sequence kernel association test^11^ (SKAT-O) is one of the most common approaches, providing a computationally efficient test that can handle scenarios wherein variants have effects in opposite directions and only a fraction of them is causal.

**Compounded:** A variant is compounded when another variant is present on the other allele. Compound heterozygotes carry two distinct mutations on the different alleles of a gene, possibly resulting in recessive disorders.

**Cytoband:** Approximate chromosomal location based on bands produced by Giemsa staining.

**Dosage sensitive:** Dosage-sensitive genes have pathogenic consequences when present in more (i.e., triplosensitive) or less (i.e., haploinsufficient) than two functional autosomal copies. Haploinsufficient genes are intolerant to heterozygous loss-of-function mutations.

**Evolutionary constraint:** Constrained genetic regions are depleted of deleterious variants as the latter are purged by natural selection. This metric indicates functionality of the region.

**Genetic interaction:** Genotype-phenotype relation depending on another factor, such as sex, environmental exposures, or other genetic variants (i.e., epistatic as opposed to additive effects).

**Healthy volunteer bias:** Type of **ascertainment bias** wherein study participants tend to be healthier and from a higher socio-economic background than the general population, affecting phenotype prevalence estimates and biasing genetic effect sizes.

**Heritability:** Fraction of phenotypic variance explained by genetic variance. Heritability can be calculated for specific sets of variants, such as rare vs. common variants, variants mapping to a specific genetic region, or belonging to a particular mutational class, to assess their contribution to phenotypes.

**Hypo-/hypermorphic alleles:** Hypomorphic alleles are partial loss-of-function alleles that result in reduced production, function, or stability of the wild-type allele. They oppose hypermorphic alleles that increase production, function, or stability of the wild-type gene product.

**Mendelian randomization:** Causal inference approach used in genetic epidemiology to identify causal relationships between two traits by leveraging genetic variants as instrumental variables.

**Penetrance and expressivity:** The penetrance of variant A for trait B describes the fraction of individuals carrying A presenting with B. If penetrance is incomplete, not all individuals exhibit the phenotype. Similarly, quantitative traits or diseases considered on a liability scale or in terms of severity of clinical presentation can have variable expressivity if not all carrier individuals of A show the same levels of B.

**Pleiotropy:** Phenomenon through which a single genetic variant or locus associates with multiple traits.

**Polygenic score (PGS):** Quantity reflecting the contribution of a group of variants to a given phenotype in a given individual. PGSs typically capture additive effects of thousands of single-nucleotide polymorphisms but can account for other mutation types or be restricted to specific genomic regions.

**Trio sequencing:** Sequencing of an affected individual (i.e., proband) and its two biological parents, allowing researchers to infer inheritance patterns (i.e., presence of a variant in parents) and identify *de novo* mutations (i.e., presence of a variant only in proband).

Box 2 **Animal models of 16p11.2 BP4-5 rearrangements**

Three series of mouse models approximate the 16p11.2 deletion (*Del/+*) or reciprocal duplication (*Dup/+*) by targeting the syntenic mouse region on chromosome 7qF3 (i.e., with conserved gene order). The first models’ rearrangement extends beyond the single-copy genes region—from *Slx1b* to *Sept1*—while at the same time excluding the ortholog of the multi-copy gene *Sult1a1*.^12^ That gene is also excluded from the second deletion mouse model.^13^ The third set of models modifies the number of copies of all genes orthologous to unique genes of the BP4-5 interval (Figure 1A), i.e., *Sult1a1* to *Spn*.^14^ However, none of these models is fully representative of the human rearrangements as the segmental duplication regions forming BP4 and BP5 are specific to *Homo sapiens*.^4^ Human deletion carrier individuals can retain multiple copies of *BOLA2/B* (MIM: 613182), *SLX1A/B* (MIM: 615822; 615823), and *SULT1A3/4* (MIM: 600641; 615819), while duplication carrier individuals harbor an even higher number of copies. For instance, deletion carrier individuals have a mode of four *BOLA2* copies, compared to six for healthy control subjects.^15^ Compounded by the poor reproducibility of mouse behavioral tests often used to proxy ASD phenotypes, differences in model engineering and/or genetic background can lead to artifactual findings. A consortium of laboratories recently set out to replicate their findings across the three deletion models, highlighting divergences across models despite globally concordant conclusions.^16^ Recent engineering of two series of rat models that delete and reciprocally duplicate the *Sult1a1*-*Spn* interval opens the possibility of studying the 16p11.2 BP4-5 CNVs in outbred rodent models (Sprague Dawley and Long Evans).^17^^,^^18^

Another approach to study the CNV is to target individual genes. The International Mouse Phenotyping Consortium (IMPC)^19^ produced and phenotyped knockout mice for 24 genes spanning the region and flanking breakpoints (Table S1). Detailed neuroanatomical phenotypes were further assessed^20^ and a similar screen in zebrafish^21^ revealed that many overlapping genes are required for proper nervous system development. While a comprehensive description of all animal models individually knocked-down for 16p11.2 BP4-5 orthologs falls out of the scope of this review, many single genes models partially replicate phenotypes observed in human 16p11.2 BP4-5 CNV carrier individuals. Furthermore, multiple studies explored double or triple hemi-deletion and their reciprocal triplosensitivity in *Drosophila*,^22^ zebrafish,^18^^,^^23^^,^^24^^,^^25^ and mice.^20^^,^^26^^,^^27^

Studies in clinical cohorts estimate the prevalence of 16p11.2 BP4-5 deletions and duplications to 1 in 360 and 1 in 390, respectively (Table 1). Hinting at their stronger deleteriousness, clinical studies found higher global **penetrance** (Box 1) for the deletion (47%) compared to the duplication (28%),^29^ as well as a higher fraction of *de novo* (as opposed to inherited) deletions (60%–90%) compared to duplications (20%–25%).^30^^,^^31^^,^^32^^,^^33^ Unlike other CNVs linked to genomic disorder that occur more frequently on the paternal haplotype, *de novo* 16p11.2 BP4-5 CNVs exhibit up to 90% maternal transmission bias which can be explained neither by older maternal age nor by imprinting, suggesting that 16p11.2 BP4-5 is a female-specific recombination hotspot.^29^^,^^31^ At the phenotypic level, 16p11.2 BP4-5 CNVs were established as an important susceptibility risk factor for autism spectrum disorders (ASD),^34^^,^^35^^,^^36^^,^^37^ developmental delay and intellectual disability,^32^^,^^38^^,^^39^ schizophrenia (SCZ),^40^^,^^41^ and seizure disorders.^39^^,^^42^^,^^43^ Additionally, mirror effects on body mass index (BMI)^44^^,^^45^^,^^46^ and head circumference^43^ were described, with deletion carrier individuals presenting with obesity and macrocephaly, while duplication carrier individuals tended to be underweight and microcephalic.

**Table 1:** Prevalence estimates of 16p11.2 BP4-5 CNVs by ascertainment strategy

**Cohort description**							**Deletion**		**Duplication**	
**Name**	***N***	**Age**	**Female**	**Country**	**Relatives**	**Ascertainment**	***n***	**Prevalence**	***n***	**Prevalence**
**Prenatal cohorts**										

West China Second University Hospital^47^	86,035	prenatal	N/A	China	N/A	pregnant women undergoing amniocentesis due to abnormal ultrasound, high-risk pregnancy, or family history of developmental delay and intellectual disability	55	0.064% (1/1,600)	N/A	N/A
Maternal and Child Health Hospital of Hubei^48^	8,578	prenatal	N/A	China	yes		17	0.198% (1/500)	4	0.047% (1/2,100)
Chengdu Women’s and Children’s Central Hospital^49^	7,078	prenatal	N/A	China	N/A		3	0.042% (1/2,400)	4	0.057% (1/1,800)
**TOTAL**	**101,691**						**75**	**0.073% (1/1,400)**	**8**	**0.051% (1/2,000)**

**Clinical cohorts**										

Baylor Genetics Laboratories^50^	54,407	pediatric	N/A	USA	no	developmental delay and intellectual disability, ASD, congenital anomalies	186	0.342% (1/290)	136	0.250% (1/400)
iPSYCH2012^51^	35,955	pediatric & young adults	43%	Denmark	yes	depression, ASD, bipolar disorder, SCZ, ADHD (born 1981–2005)	28	0.078% (1/1,300)	88	0.245% (1/410)
Signature Genomics Laboratories^29^	33,226	pediatric	N/A	USA (mainly EUR)	N/A	developmental delay and intellectual disability, epilepsy, ASD, congenital anomalies, dysmorphic features	146	0.439% (1/230)	93	0.280% (1/360)
Epi25 Collaborative^52^	26,699	N/A	N/A	international (92% EUR)	N/A	seizure and epilepsy disorders	44	0.165% (1/610)	34	0.127% (1/790)
Psychiatric Genomics Consortium (PGC) - SCZ^53^	21,094	adult	N/A	international (100% EUR)	no	SCZ	N/A	N/A	70	0.332% (1/300)
SCZ meta-analysis^54^	9,384	adult	44%	China	N/A	SCZ	N/A	N/A	26	0.277% (1/360)
ADHD meta-analysis^55^	8,883	pediatric & adult	43%	Iceland, Norway	yes	ADHD	7	0.079% (1/1,270)	17	0.191% (1/520)
CLOZUK1+2^56^	6,934	adults	29%	UK	N/A	treatment-resistant SCZ	4	0.058% (1/1,700)	47	0.678% (1/150)
Developmental delay and intellectual disability meta-analysis^38^	4,284	pediatric	N/A	international (mainly EUR)	yes	developmental delay and intellectual disability or congenital anomalies	22	0.514% (1/200)	N/A	N/A
Children’s Hospital Boston^57^	3,450	pediatric	N/A	USA	N/A	developmental delay and intellectual disability, ASD, dysmorphic features, congenital anomalies	20	0.580% (1/170)	N/A	N/A
Obesity meta-analysis^44^	3,103	pediatric & adult	N/A	Europe	N/A	obesity	26	0.838% (1/120)	0	0%
Chronic Kidney Disease in Children (CKiD) + KIdney of MONofunctional Origin (KIMONO)^58^	2,824	pediatric & young adults	43%	international (90% EUR)	no	congenital anomalies of the kidney and urinary tract	7	0.248% (1/400)	1	0.035% (1/2,800)
Bipolar Disorder Research Network (BDRN)^59^	2,591	adults	69%	UK	no	bipolar disorder	N/A	N/A	3	0.116% (1/860)
Autism Genetic Resource Exchange (AGRE) + Autism Case-Control (ACC)^60^	2,195	pediatric & young adults	20%	USA (mainly EUR)	yes	ASD	9	0.410% (1/240)	8	0.364% (1/270)
ASD cohort^61^	1,132	pediatric & young adults	22%	Japan	N/A	ASD	1	0.088% (1/1,100)	4	0.353% (1/280)
Simons Simplex Collection (SSC)^62^	1,124	pediatric & young adults	14%	USA (75% EUR)	no	ASD	8	0.712% (1/140)	6	0.534% (1/190)
**TOTAL**	**217,285**						**508**	**0.276% (1/360)**	**533**	**0.254% (1/390)**
**Meta-analysis (any disease)**^63^								**0.264%**		**0.153%**

**Population-based cohorts**										

UK Biobank^64^	331,522	adults	54%	UK	no	recruited from the general population through invitation (born 1936–1970)	73	0.022% (1/4,500)	89	0.027% (1/3,700)
deCODE genetics^55^	155,122	adults	54%	Iceland	yes	recruited from the general population	56	0.036% (1/2,800)	69	0.044% (1/2,200)
DiscovEHR^65^	90,595	adults	61%	USA (98% EUR)	yes	recruited through health care system	59	0.065% (1/1,500)	63	0.070% (1/1,400)
Estonian Biobank^64^	89,516	adults	66%	Estonia	no	recruited from the general population by general practitioner/hospital physicians	14	0.016% (1/6,400)	11	0.012% (1/8,100)
Bio*Me*^66^	24,877	adults	59%	USA (32% EUR)	yes	recruited through health care system	15	0.060% (1/1,700)	4	0.016% (1/6,200)
FINRISK^67^	23,053	adults	53%	Finland	no	representative sample of ∼2,000 individuals in each Finnish region collected every 5 years (1992–2012)	6	0.026% (1/3,800)	5	0.022% (1/4,600)
Rosenfeld et al. controls^29^	22,246	adults	N/A	international (mainly EUR)	N/A	neurologically normal adults from various cohorts	6	0.027% (1/3,700)	9	0.040% (1/2,500)
iPSYCH2012 controls^51^	19,169	pediatric & young adults	49%	Denmark	yes	random individuals (born 1981–2005)	10	0.052% (1/1,900)	21	0.110% (1/910)
Norwegian Mother, Father and Child Cohort Study (MoBA)^68^	12,252	newborns	N/A	Norway	yes	children from volunteer pregnant women attending routine ultrasound (born 1999–2009)	6	0.049% (1/2,000)	5	0.041% (1/2,500)
NFBC1966^67^	4,895	newborns	49%	Finland	no	Norther Finland Birth Cohort: all children born in Oulu and Lappland (1966).	3	0.061% (1/1,600)	3	0.061% (1/1,600)
**TOTAL**	**773,247**						**248**	**0.032% (1/3,100)**	**279**	**0.036% (1/2,800)**
**Meta-analysis (no disease)**^63^								**0.026%**		**0.032%**

Prevalence of 16p11.2 BP4-5 deletion and duplication estimated from non-overlapping cohorts with different ascertainment strategies: prenatal, clinical, and population cohorts. The cohort description includes the cohort’s name/sample origin and the reference from which data were retrieved. *N* indicates cohort sample size. Age indicates the predominant age group. Female indicates the proportion of females in the full cohort. Country indicates where samples were recruited. For most cohorts, the predominant ancestry group matches the most common ancestry group of the recruitment country. For USA and international cohorts, the proportion of individuals of European (EUR) ancestry is indicated when available. The relatives column specifies if relatives are present or not in the cohort. Ascertainment describes how participants were recruited. The number of carrier individuals (*n*) and prevalence of the deletion and duplication are reported. N/A indicates that data were not reported. Average prevalence is calculated for each ascertainment strategy. For clinical and population cohorts, these numbers are put in comparison to the prevalence of carrier individuals among individuals with at least one or no diseases in a large meta-analysis (samples might overlap with some of the reported cohorts).^63^ ADHD, attention-deficit hyperactivity disorder; ASD, autism spectrum disorder; SCZ, schizophrenia.

## Beyond clinical cohorts

Large biobanks allowed estimating the prevalence of 16p11.2 BP4-5 deletions and duplications in the general population to 1 in 3,100 and 1 in 2,800, respectively, corresponding to 8-fold lower estimates than in clinically ascertained cohorts (Table 1). Interestingly, the largest CNV meta-analysis to date estimated 16p11.2 BP4-5 CNV frequency in ∼1 million individuals, splitting their samples according to whether they were diagnosed or not with any of a broad range of 54 diseases.^63^ While the former group’s prevalence aligned with our clinical cohort’s prevalence estimate, the latter aligned with our population cohort estimate (Table 1). Furthermore, our deletion frequency estimate for population cohorts matches the 1 in 3,021 predicted by another study based on clinical and epidemiological data.^50^ Some cohorts, such as Bio*Me*,^66^ exhibit stronger differences in deletion versus duplication prevalence, possibly due to its healthcare cohort enrollment protocol. This showcases the role of **ascertainment bias** (Box 1) in obtaining accurate prevalence estimates. While clinical cohorts are enriched for CNV carrier individuals, population studies suffer from a **healthy volunteer bias**^69^ (Box 1), leading to prevalence underestimation. Prenatal cohorts, which are less biased in their ascertainment, yield intermediate prevalence estimates (Table 1), suggesting that true prevalence lies in between estimates from clinical and population cohorts. Nevertheless, presence of carrier individuals in cohorts largely considered to be healthy reinforces a model of incomplete penetrance and variable expressivity. Because biobanks are typically coupled with comprehensive phenotypic assessment and electronic health records, they offer the opportunity to evaluate the consequences of 16p11.2 BP4-5 CNVs in older populations that are not ascertained for severe clinical conditions and probably are at the milder end of the phenotypic spectrum.

Besides replicating core features associated with 16p11.2 BP4-5 CNV carrier individuals, such as decreased cognitive ability^70^^,^^71^^,^^72^ or the mirror effect on BMI,^73^ phenome-wide analyses in population studies consistently highlighted 16p11.2 BP4-5 as one of the most **pleiotropic** (Box 1) structural rearrangements genome-wide.^63^^,^^64^^,^^66^^,^^74^^,^^75^^,^^76^^,^^77^^,^^78^ We recently developed a framework to perform CNV genome-wide association studies (GWASs) in the UK Biobank (UKBB), allowing us to assess the impact of 16p11.2 BP4-5 CNVs on 117 complex traits and diseases according to four dosage mechanisms^64^^,^^78^ (Figure 2B). A total of 46 traits were significantly affected by CNVs in the region^28^ (Figure 2C). Deletions were more deleterious, leading on average to 2.8 additional disease diagnoses (*p* = 2.6 × 10^−24^), as opposed to 0.3 for duplication carrier individuals (*p* = 0.183). About 9% of the signals were better captured by a U-shape model, including those related to cognitive function and grip strength. Conversely, 22% of the associations exhibited a mirror effect on puberty timing, liver enzymes, heel bone mineral density, or sleep apnea risk. The marked difference between the U-shaped and mirror models indicates that disparate evolutionary forces (e.g., directional vs. stabilizing selection) may act on the expression level of genes in the region. Most importantly, and in line with the syndromic nature of 16p11.2 BP4-5 rearrangements, associations involved a broad spectrum of physiological systems, even after accounting for potential confounders such as adiposity levels^28^ (Figure 2C).

Here, we review evidence from both clinical and population studies to describe the full phenotypic spectrum associated with 16p11.2 BP4-5 CNVs. Highlighting the complementarity of these approaches, we further discuss the importance of awareness around phenotypic heterogeneity and adoption of diverse data sources and analytic strategies to better diagnose, monitor, and possibly prevent 16p11.2 BP4-5-associated comorbidities.

## Pleiotropy of 16p11.2 rearrangements

### Psychiatry

#### Autism spectrum disorder

ASD was associated with 16p11.2 BP4-5 CNVs in the late 2000s and likely represents one of the best-studied phenotypic consequences of the rearrangement. This is notably due to efforts aiming at building large cohorts of individuals with ASD (Box 3), which found that about 1% of individuals with the disorder carry the deletion, while another 1% carry the duplication,^34^^,^^35^^,^^36^^,^^37^^,^^79^ making 16p11.2 BP4-5 CNVs one of the strongest genetic risk factors for ASD. About 20% of individuals carrying a 16p11.2 BP4-5 CNVs show autistic features,^80^^,^^81^^,^^82^^,^^83^ so that the CNV is commonly used as a model to study the disease.^84^ For example, 22% and 26% of 217 and 114 16p11.2 deletion and duplication carrier individuals presented with ASD, respectively, with a wider variation for psychiatric disorder for duplication carrier individuals.^82^ Accordingly, the role of the CNV in ASD and other neurodevelopmental phenotypes has been reviewed previously.^85^^,^^86^^,^^87^ From a mechanistic point of view, overall sensory processing is affected in deletion carrier individuals^88^^,^^89^^,^^90^ and numerous changes in brain structure and function have been described (see structural and functional alterations of the nervous system). Targeted and ASD **trio sequencing** (Box 1) identified *de novo* and/or potentially causative variants in six genes from the 16p11.2 BP4-5 interval: *MAZ* (1 case [MIM: 600999]), *SEZ6L2* (2 cases [MIM: 616667]), *TAOK2* (5 cases [MIM: 613199]), *KCTD13* (2 cases [MIM: 608947]), *MAPK3* (9 cases [MIM: 601795]), and *CORO1A* (2 cases [MIM: 605000]).^23^^,^^91^^,^^92^^,^^93^^,^^94^^,^^95^^,^^96^^,^^97^^,^^98^^,^^99^ Consistent with the CNV’s pleiotropy, similar mutations were identified in some of the same, as well as other genes in the interval in developmental delay and intellectual disability cases—*TAOK2* (2 cases), *MAPK3* (5 cases), *MVP* (1 case [MIM: 605088]), and *DOC2A* (1 case [MIM: 604567])^99^^,^^100^^,^^101^—and SCZ trios—*KCTD13* (1 case) and *TMEM219* (1 case [MIM: 620290]).^102^^,^^103^ The involvement of some of these genes in ASD was further supported by dedicated experiments.^104^^,^^105^^,^^106^^,^^107^ For example, the protein encoded by the immunity and platelet biology gene *CORO1A*^108^^,^^109^ is part of the AP2-mediated clathrin-coated pit subcomplex within the atlas of autism protein interactions.^110^ Taken together, this suggests that contrary to other genomic disorders (e.g., 17q21.31 Koolen-De Vries syndrome [MIM: 610443]), there does not seem to be a single major phenotypic driver for neurodevelopmental phenotypes within the interval and interaction with nearby regions on the short arm of chromosome 16p were shown to additionally contribute to ASD risk.^111^^,^^112^ Paralleling human findings, response to social cues is correspondingly altered in mouse^113^^,^^114^ and rat^17^ deletion models. Similarly, mouse models of the duplication present with social and cognitive deficits that coincide with electrophysiological perturbations in brain regions involved in these functions.^115^^,^^116^

Box 3 **Cohorts of 16p11.2 BP4-5 CNV carrier individuals**

The strong link between 16p11.2 BP4-5 CNVs and ASD has motivated genotype-first approaches to elucidate the pathological mechanisms of the disease, leading to the creation of the first cohorts of 16p11.2 CNV carrier individuals: the Simons Variation in Individuals Project (Simons VIP; now part of Simons Searchlight)^84^ and the 16p11.2 European Consortium.^81^ The bulk of our current knowledge of the rearrangement stems from the relatively small set of individuals enlisted in these cohorts. More recently, the impact of the CNV has been assessed in large population cohorts such as the UK Biobank^117^ and the Estonian Biobank,^118^ allowing researchers to study a larger pool of carrier individuals with increased diversity in terms of CNV expressivity. For instance, efforts from the Enhancing NeuroImaging Genetics through Meta-Analysis CNV (ENIGMA-CNV) group aim to meta-analyze brain imaging data from both population and clinical CNV carrier individuals.

While these cohorts have pioneered the field, they are mainly composed of individuals of European ancestry. More diverse population cohorts have been set up in recent years,^119^^,^^120^^,^^121^^,^^122^ allowing researchers to better grasp the extent to which frequency and phenotypic expression depend on an individual’s ancestral background (see diversity in ancestry).^123^

#### Schizophrenia and psychosis

Shortly after describing the association with ASD, the 16p11.2 BP4-5 duplication—but not its deletion—was identified as a major risk factor for SCZ.^41^ This association was replicated multiple times,^53^^,^^56^^,^^124^ including in individuals of Han Chinese ancestry,^54^ leading to a 10-fold increase in SCZ risk with a penetrance of 7%.^125^ Accordingly, duplication carrier status also increases risk for psychotic symptoms,^126^ a hallmark of SCZ. These results contrast with recent results from the Danish Lundbeck Foundation Initiative for Integrative Psychiatric Research (iPSYCH), which did not find any significant effect of 16p11.2 BP4-5 CNVs on SCZ risk.^127^ This study also found a damped effect for other SCZ CNVs, e.g., 22q11.2 deletion, suggesting that these results stem from differences in ascertainment. Compatible with a model wherein both the deletion and duplication increase risk for SCZ, 4.1% of deletion and 4.6% of duplication carrier individuals are diagnosed with the disease in UKBB.^78^ While never meeting criteria for significance, deletion carrier individuals have been identified in SCZ clinical cohorts.^41^^,^^53^^,^^56^^,^^124^ We hypothesize that milder affliction of deletion carrier individuals in population cohorts unmasks SCZ, whose diagnosis is impaired in clinically ascertained deletion carrier individuals with severe developmental delay and/or intellectual disability.

#### Other psychiatric conditions

Over the last 15 years, the pleiotropic effect of 16p11.2 BP4-5 CNVs on psychiatric conditions became increasingly evident.^51^^,^^80^^,^^81^^,^^82^^,^^83^^,^^127^^,^^128^ On average, clinically ascertained deletion carrier individuals were diagnosed with 2.9 psychiatric conditions, a 10-fold increase compared to familial control subjects.^128^ For instance, attention-deficit hyperactivity disorder (ADHD) was consistently reported in descriptive studies of clinically ascertained 16p11.2 BP4-5 CNV carrier individuals, affecting up to 30% of carrier individuals,^30^ with slightly higher prevalence among duplication carrier individuals.^32^^,^^43^^,^^82^^,^^83^^,^^128^^,^^129^ The link between the duplication and ADHD was confirmed in 8,883 affected individuals of Icelandic and Norwegian origin,^55^ as well as in iPSYCH.^51^^,^^127^ A nominally significant association with ADHD remains upon exclusion of ASD and SCZ affected individuals,^55^ indicating that the condition can arise independently of the latter diagnoses. The 16p11.2 BP4-5 duplication also represents the only CNV robustly associated with the risk of bipolar disorder in clinical cohorts,^59^^,^^41^ an association confirmed in UKBB,^78^ where at least 9% of duplication carrier individuals are diagnosed with the condition. A single study reported a nominally significant enrichment for 16p11.2 BP4-5 CNV carrier individuals among 604 individuals with major depressive disorder.^130^ In the UKBB, the 16p11.2 BP4-5 duplication was identified as one of three recurrent CNVs associated at Bonferroni significance level with self-reported depression, even after excluding individuals with other neuropsychiatric conditions.^131^ These results were replicated based on hospital-diagnosed individuals in UKBB^78^ but not in iPSYCH,^51^^,^^127^ paralleling the dampened effect size observed for SCZ. 16p11.2 BP4-5 CNVs further have been linked to anxiety, disruptive behavior, tic disorders, and obsessive-compulsive disorders,^65^^,^^81^^,^^82^^,^^83^^,^^126^^,^^128^^,^^132^^,^^133^ although with more limited evidence. Perplexingly, neuroticism, which strongly correlates with several psychiatric conditions,^134^ was associated with neither duplication nor deletion carrier status in UKBB.^64^ While both duplications and deletions are now recognized as important risk factors for psychiatric conditions, current evidence suggests higher prevalence and heterogeneity in diagnoses among duplication carrier individuals.^82^^,^^83^ In line with this, psychiatric conditions are the only disease category primarily driven by the region’s duplication in UKBB.^78^ Further research is required to delineate the precise nature and penetrance of distinct psychiatric disorders linked to 16p11.2 BP4-5 rearrangements and to explore shared disease mechanisms.

### Neurology

#### Developmental delay and intellectual disability

Developmental delay and intellectual disability were present in virtually all probands of early descriptive studies of 16p11.2 BP4-5 CNV carrier individuals^32^^,^^39^^,^^43^ and clinical cohorts ascertained for the latter diagnoses were systematically enriched for 16p11.2 BP4-5 CNV, and particularly deletion, carrier individuals.^38^^,^^135^ Compared to non-carrier parents, deletion probands have an average reduction of 25–35 full-scale intelligence quotient (IQ) points^81^^,^^136^—with similar findings for duplication carrier individuals^80^^,^^137^—so that one-third of clinically ascertained CNV carrier individuals meet intellectual disability criteria.^82^ Duplication carrier individuals exhibit higher variation in full-scale IQ, with an almost 20-fold enrichment for individuals with extremely low values, compared to deletion carrier individuals.^80^ Reduced cognitive performance was replicated in multiple population cohorts,^70^^,^^71^^,^^72^ including the UKBB, where a significant U-shape effect on fluid intelligence score was observed with a slightly stronger effect in duplication carrier individuals.^28^^,^^64^ Together, this makes developmental delay and intellectual disability among the most consistently associated traits with the region’s rearrangement.

One crucial component of developmental delay in 16p11.2 BP4-5 CNV carrier individuals is **language and speech impairment**^30^^,^^32^^,^^36^^,^^38^^,^^39^^,^^43^^,^^128^ (Box 4), which manifests through lower verbal IQ and high (83%) rates of speech and language therapy during childhood among deletion carrier individuals.^81^ All language components are negatively impacted in deletion carrier individuals, with milder evidence in duplication carrier individuals.^138^^,^^139^ Motor speech disorders are common, with 79% of deletion and 30% of duplication carrier individuals suffering from speech articulation defects,^133^ possibly due to reduced sensorimotor adaptation.^140^ There is evidence that the 16p11.2 BP4-5 deletion predisposes to childhood apraxia of speech,^141^^,^^142^^,^^143^ a rare motor speech condition affecting planning and coordination of movements required for speech. The latter diagnosis often co-occurs with receptive (73%) and expressive (70%) language disorders, as well as mild-to-moderate speech impairments (89%).^143^ If about three-quarters of children carrying a deletion meet childhood apraxia of speech diagnostic criteria, two-thirds of them go undiagnosed^143^ and prevalence estimates among duplication carrier individuals are currently lacking. Presence of cognitive delay or ASD exacerbates speech and language impairment but cannot fully account for them, indicating that the latter represents a core feature of the 16p11.2 BP4-5 rearrangement, with exacerbated penetrance in deletion carrier individuals.^138^^,^^139^^,^^143^ No clear candidate gene has been established for speech and language phenotypes but a recent study in 50,000 individuals found that loss of function of the 16p11.2 gene *MAZ* associated at nominal significance with stuttering.^144^

Box 4 **Medical glossary**

**Absence epilepsy:** More frequent in children, the generalized onset seizures of absence epilepsy are characterized by very brief, sudden-onset periods of “blanking out” and often disappear in adolescence.

**Chiari type I malformation:** Cerebellar herniation in the spinal canal due to skull malformation (or small skull). Type I malformations are the least severe ones.

**Childhood epilepsy with centrotemporal spikes:** Formerly known as Rolandic epilepsy, it is the most common form of epilepsy in childhood and is characterized by seizures originating in the Rolandic area of the brain. Seizures usually disappear in adolescence.

**Craniosynostosis:** Rare birth defect characterized by premature fusion of skull bones that can affect brain development.

**Developmental and epileptic encephalopathies (DEEs):** Group of rare and severe epileptic syndromes characterized by severe seizures and epileptic activity that leads to cognitive impairment/regression. DEEs are often refractory to treatment and associated with early age of onset.

**Language and speech impairment:** Language disorders describe difficulties in understanding (receptive language) or getting across (expressive language) a message. They are subdivided into phonology, lexicon, syntax, semantics, and pragmatics. Speech disorders refer to conditions impairing the formation of the sounds necessary to communicate.

**Müllerian aplasia:** Also known as Mayer-Rokitansky-Küster-Hauser syndrome, Müllerian aplasia is a rare congenital defect of the female reproductive system characterized by aplasia of the uterus, cervix, and vagina, leading to infertility. It can co-occur with malformations of the Fallopian tubes, ovaries, urinary tract, and spine, in which case it is referred to as Müllerian-renal-cervicothoracic somite dysplasia.

**Posterior fossa:** Small cavity in the skull in which the cerebellum and part of the brain stem are located. Malformations typically affect cerebellum development and are classified depending on whether the fossa is enlarged (e.g., Dandy-Walker malformation) or too small (e.g., rhombencephalosynapsis).

**Self-limited familial and non-familial infantile epilepsy (SeLIE)**: Formerly known as benign infantile seizures, SeLIE seizures typically start around 6 months and remit within one year of onset, without disrupting developmental progress.

**Spondylocostal dysostosis:** Rare disorder characterized by severe, congenital deformities of the spine and ribs that cause short-trunk dwarfism. Deformities increase risk of breathing problems, hernia, spina bifida, and **Chiari malformations**.

**Vesicoureteral reflux:** Abnormal flow of urine from the bladder back up the ureters toward the kidneys, which increases infection risk and can cause renal damage.

Developmental trajectories in childhood are globally similar between deletion and duplication carrier individuals, with an increase in verbal IQ over time.^129^ Concerning motor function, 47%–67% of deletion and 22%–56% of duplication carrier individuals get diagnosed with developmental coordination disorder.^129^^,^^145^ Motor delays include feeding difficulties in newborns,^43^ hypotonia,^32^^,^^133^ hyporeflexia,^133^ poor agility,^133^ late age at first walking (>15 months),^80^^,^^145^ and impaired balance, speed, and endurance in locomotion tests.^146^ If most of these are observed in all CNV carrier individuals, duplication carrier individuals showed stronger impairments with additional features such as hyperreflexia (32%) and tremors (43%),^133^ as well as very late onset walking.^80^ Duplication carrier individuals also have worse accuracy and speed in a battery of neurocognitive assessments evaluating executive function, episodic memory, complex and social cognition, and psychomotor speed, compared to deletion carrier individuals.^147^ Furthermore, diagnosed deletion (*n* = 48) and duplication (*n* = 48) carrier individuals in the Vanderbilt University Medical Center’s biobank (BioVU) showed increased rates of “abnormal movement and developmental delay” (CNV carrier individuals), “muscle weakness” (deletion carrier individuals), and “speech and language disorders” (duplication carrier individuals) in their electronic health records.^148^ Adult populations are not ideally suited to study language, speech, and motor impairment, and only a very few diagnosed cases of language and speech, scholastic skills, and motor impairment are present in UKBB. Yet, decreased grip strength was observed in both deletion and duplication carrier individuals,^64^ suggesting that impaired motor function persists in adulthood.

#### Structural and functional alterations of the nervous system

Recent efforts have concentrated on identifying brain alterations that could explain the predisposition of 16p11.2 BP4-5 CNV carrier individuals for neurodevelopmental and psychiatric disorders. One striking feature includes the global increase of brain size—including total intracranial, white matter, and gray matter volumes—among deletion carrier individuals, which opposes the pervasive size reduction observed among duplication carrier individuals^149^^,^^150^^,^^151^^,^^152^ and aligns with the previously described macrocephaly and microcephaly phenotypes observed in deletion and duplication carrier individuals, respectively^41^^,^^43^ (see craniofacial features). Changes in brain volume have been modeled in cellular models and cortical organoids, i.e., 3D cell cultures derived from embryonic or induced pluripotent stem cells aiming to partially recapitulate brain structure and organization, as well as cell-cell interactions. These models show that dosage negatively correlates with neuron size, dendrite length, and neuronal differentiation.^153^^,^^154^^,^^155^ Focal cortical anomalies are widespread among CNV carrier individuals. They correlate negatively with full-scale IQ, with duplication carrier individuals exhibiting an increased number of abnormally thin cortex areas, while deletion carrier individuals exhibit increased cortical thickness.^156^ Up to a quarter of duplication carrier individuals present with increased ventricular volume^152^^,^^157^ and cerebellar tonsillar ectopia or **Chiari type I malformations** (Box 4) (MIM: 118420) have been reported in up to a third of deletion carrier individuals.^80^^,^^81^^,^^133^^,^^157^^,^^158^^,^^159^ These alterations are often present at age 5 and remain stable until adulthood.^152^^,^^157^ Pointing at a prenatal age of onset, 16p11.2 BP4-5 deletions were the most common CNV in 242 fetuses with ventriculomegaly (4.5% of cases).^160^ Dosage effect on white matter microstructure was also identified,^161^ with deletion carrier individuals consistently showing increased diffusivity that could reflect decreased myelin or axonal density.^161^^,^^162^^,^^163^^,^^164^ Anomalies often involve regions involved in auditory, language, speech, and social function,^151^^,^^156^^,^^162^^,^^164^ the reward system,^150^^,^^151^ or the cerebellum,^149^^,^^151^ all of which play crucial roles in the etiology of phenotypes commonly observed among 16p11.2 BP4-5 CNV carrier individuals. At the molecular level, neuroanatomical changes have been reported in over 14 mouse models with individual 16p11.2 BP4-5 ortholog deletions^20^—including *Mapk3* (*Erk1*),^165^ *Taok2*,^91^^,^^107^ *Mvp*,^20^ and *Doc2a*^166^—often resulting in cognitive or behavioral deficits. This emphasizes that brain morphology is highly polygenic and likely regulated by multiple genes of the region.

Aligning with the idea that brain structure correlates with function, impaired prefrontal connectivity was found in human and mouse 16p11.2 BP4-5 deletion carrier individuals,^167^ with global reinforcement of functional connectivity among deletion carrier individuals and a trend for lower connectivity among duplication carrier individuals, suggesting a dosage effect.^168^ Specifically, pervasive increase in intra-axonal volume in multiple white matter tracts is already visible at an early age (2 years) in deletion carrier individuals.^169^ In parallel, several studies have reported atypical neural activity upon auditory,^170^^,^^171^^,^^172^ visual,^173^^,^^174^ or social^175^ stimuli, as well as during preparation of overt speech and hand movement, with left hemispheric language specialization being decreased among deletion carrier individuals.^176^ The deletion mouse model correspondingly showed abnormally high activity in the motor cortex during learning in males.^177^ The impact of the duplication on brain signal processing remains less clear.

Neurophysiological differences might translate into the broad spectrum of phenotypic alterations observed in 16p11.2 BP4-5 CNVs. Indeed, affected brain areas overlap with the ones altered in idiopathic psychiatric cases^151^—with a particularly strong correlation between the effect of the region’s deletion and ASD^178^—but also harbor some unique features.^149^ Importantly, 16p11.2 BP4-5 CNVs exert a stronger effect on overall brain structure^178^ and connectivity^168^ than idiopathic cases of ASD or SCZ, motivating genotype-first approaches to elucidate the pathological mechanisms of these diseases (Box 3). Interestingly, brain structure profiles defined from clinically ascertained CNV carrier individuals mimicked those of 7 duplication and 4 deletion carrier individuals with available brain imaging in the UKBB and associated with 55 and 34 traits, respectively, linking them more broadly to the human phenome.^179^ Low frequency of 16p11.2 BP4-5 CNVs compounded by the even smaller number of carrier individuals with brain imaging will make collaborative approaches crucial to establish the impact of the region’s dosage on brain structure and connectivity and interpret their functional consequences (Box 3).

#### Seizure disorders

About 10%–30% of clinically ascertained CNV carrier individuals suffer from seizure disorders and epilepsy.^30^^,^^32^^,^^38^^,^^39^^,^^43^^,^^80^^,^^81^^,^^133^^,^^180^^,^^181^ For example, a case-control study found that both deletion and duplication carrier individuals were enriched in over 26,000 individuals diagnosed with epilepsy and seizures.^52^ CNVs are associated with both severe and milder epilepsies. Two 16p11.2 BP4-5 duplication carrier individuals were identified among 315 individuals with **developmental and epileptic encephalopathies** (DEEs)^182^ (Box 4). These findings parallel case reports of duplication carrier individuals with epilepsy of infancy with migrating focal seizures,^183^ Landau-Kleffner syndrome,^180^ and epileptic encephalopathy with continuous spike and wave in sleep,^181^ as well as a deletion carrier with West syndrome.^184^ West syndrome was also diagnosed in 0.5% of 390 deletion and 1.1% of 270 duplication carrier individuals.^80^ Milder epilepsies, such as **childhood epilepsy with centrotemporal spikes** (Box 4) (MIM: 117100), was diagnosed in 1.5% of duplication carrier individuals,^180^ a finding supported by a smaller study identifying two duplication carrier individuals among 47 cases.^185^ This association was specific to duplication carrier individuals, who were not enriched for other epilepsy types.^180^ Conversely, **absence epilepsy** (Box 4) was observed in 33% of deletion carrier individuals (versus 5% of duplication carrier individuals).^133^ A systematic characterization of seizure disorders among 16p11.2 BP4-5 CNV carrier individuals found that **self-limited familial and non-familial infantile epilepsy (SeLIE)** (Box 4) (MIM: 605751) was the most common seizure disorder among deletion carrier individuals, accounting for 42% of epilepsies^181^ and was found in 3 out of 33 deletion carrier individuals in a Dutch study.^186^ SeLIE accounted for only 13% of epilepsies among duplication carrier individuals, which presented with a more heterogeneous disease spectrum,^181^ paralleling the trend described for other neuropsychiatric conditions. While we previously reported increased epilepsy risk among UKBB deletion carrier individuals,^78^ the association falls below the threshold for significance in a re-analysis.^28^ Overall, 16p11.2 BP4-5 CNVs contribute to a broad spectrum of epileptic disorders with varying degrees of severity, with the region’s dosage affecting epilepsy subtype. Consistent with this hypothesis, the 16p11.2 genes *PRRT2* (MIM: 614386) and *SEZ6L2* act as hubs in an epilepsy protein subnetwork dysregulated in a duplication mouse model and correcting the dosage of *PRRT2* rescued seizure susceptibility.^187^ In zebrafish, an epistatic contribution to seizure susceptibility has been reported in double *doc2a*^*+/−*^*fam57b4*^*+/−*^ knockdowns,^24^ suggesting oligogenic contribution to the phenotype.

#### Movement disorders

Paroxysmal kinesigenic dyskinesia (PKD) (MIM: 128200), a rare movement disorder characterized by brief and recurrent involuntary movement attacks, has been associated with 16p11.2 BP4-5 deletions.^188^^,^^189^^,^^190^^,^^191^^,^^192^ PKD can co-occur with SeLIE, a combination of features referred to as infantile convulsion with choreoathetosis syndrome (ICCA) (MIM: 602066). These disorders were shown to be caused by heterozygous variants in *PRRT2*.^187^^,^^193^ In a review of 1,444 published cases with 70 distinct *PRRT2* mutations, 42%, 39%, and 14% of affected individuals were diagnosed with SeLIE, PKD, and ICCA, respectively, with the remaining affected individuals suffering from various disorders, including seizures and headache disorders.^193^ Importantly, *PRRT2* mutations can lead to different disorders within the same family.^194^ SeLIE is typically not associated with neurodevelopment outcomes, but sudden and extreme autistic regression was reported in a 15-month-old female with SeLIE carrying a heterozygote deleterious *PRRT2* variant.^195^ While the pleiotropy and variable expressivity of *PRRT2* haploinsufficiency are well established, further research is required to understand how it relates to the 16p11.2 BP4-5 deletion pleiotropy.

### Endocrinology and metabolism

#### Obesity

Obesity was frequent among the first described 16p11.2 BP4-5 deletion carrier individuals^38^^,^^36^^,^^37^^,^^39^^,^^196^ but was recognized as a core feature of the rearrangement only when 1%–3% of individuals suffering from severe obesity were found to carry the deletion,^45^^,^^46^ an association reproduced in large clinical cohorts.^81^^,^^197^ While feeding difficulties and failure to thrive have been reported early in life,^197^ BMI was consistently found to increase at around 4–6 years and rapidly progresses to obesity,^46^^,^^197^^,^^198^^,^^199^ with a penetrance of 70% in adults.^81^ Conversely, duplication carrier individuals are at increased risk for being underweight, establishing a negative correlation between the region’s dosage and BMI and demonstrating for the first time that overweight and underweight could have the same etiology.^44^^,^^80^ This mirror effect was replicated in UKBB, with the deletion and duplication leading to a BMI increase and decrease of 6.2 kg/m^2^ and 1.8 kg/m^2^, respectively.^73^ Similar findings in population cohorts have since been reported for continuous measures of adiposity such as BMI, weight, or body fat mass,^64^^,^^74^^,^^75^^,^^200^ as well as binary diagnosis of obesity.^76^^,^^200^ Multiple studies also reported an increase in waist-to-hip ratio,^64^^,^^73^^,^^74^ indicative of a shift from subcutaneous to visceral adiposity that has been linked to adverse health outcomes, even if this association is strongly attenuated upon adjustment for BMI.^64^ Hyperphagia is prevalent among deletion carrier individuals, especially those suffering from obesity^30^^,^^36^^,^^46^^,^^81^ and deletion carrier individuals also exhibit altered satiety response preceding obesity onset,^201^ as well as structural changes in brain areas associated with reward mechanisms.^150^^,^^151^ Consistent with this observation, deletion carrier individuals are prone to disinhibiting eating disorders with eating in the absence of hunger when they see others eat or when they are bored, even if this behavior cannot fully account for BMI increase.^198^ This suggests that other mechanisms are also at play, e.g., motor delays and slower walking pace^75^ impairing capacity to exercise, thereby reducing energy expenditure. Overall, the abundance of evidence from clinical and population cohorts makes the dosage effect on BMI one of the most striking and robust features linked to 16p11.2 BP4-5 CNVs.

#### Other features of the metabolic syndrome and obesity-related comorbidities

Despite obesity representing a major risk factor for numerous diseases, few clinical studies have investigated other features of the metabolic syndrome among CNV carrier individuals, so that most of current knowledge stems from adult population cohorts. Our recent UKBB study found that the association between 16p11.2 BP4-5 CNVs and 22 phenotypes was lost upon conditioning on BMI (Figure 2C), with Mendelian randomization supporting a causal mediatory role of BMI for most of them.^28^ For instance, increased risk for hypertension, altered serum lipid levels, and elevated levels of the inflammation marker C-reactive protein (CRP) and serum urate levels were secondary to the deletion’s impact on adiposity (Table 2). Conversely, increased levels in glycated hemoglobin and hepatic biomarkers were at least partially independent from the deletion’s impact on BMI (Table 2), suggesting that other mechanisms could promote risk for type 2 diabetes and liver disease. This parallels findings for the adjacent BP2-3 deletion^202^ (Figure 1A), but more research is needed to decipher underlying molecular mechanisms. Interestingly, in mouse models for the CNV, the mirror effect is reversed,^12^^,^^13^^,^^14^ with the deletion leading to small body size and altered basal metabolism,^203^ while the duplication causes severe weight gain, hepatic steatosis, hyperlipidemia, and hyperinsulinemia.^204^ Mice with the deletion further exhibit altered brain metabolism and reduced number of mitochondria in brain endothelial cells.^205^ Using human and zebrafish models, haploinsufficiency of the ceramide synthase modulator *TLCD3B* (previously *FAM57B* [MIM: 615175]) was shown to disrupt sphingolipid and glycerolipid homeostasis in the brain, leading to defects in synaptogenesis, brain activity, and behavior.^206^ While several studies have suggested that obesity is independent of the neuropsychiatric phenotypes frequently observed among CNV carrier individuals,^46^^,^^80^^,^^81^^,^^201^ these new reports hint at a potential link between metabolic and neurologic phenotypes.^205^^,^^206^

**Table 2:** Metabolic syndrome features associated with 16p11.2 BP4-5 CNVs in the UK Biobank

**Phenotype**	**Current evidence**	**Effect of BMI conditioning**^28^
Type 2 diabetes	deletion carrier individuals are at increased risk for type 2 diabetes^75^^,^^76^^,^^200^ and exhibit higher levels of glycated hemoglobin^64^^,^^75^^,^^77^	association with glycated hemoglobin is partially independent of BMI
Hypertension	deletion carrier individuals are at increased risk for essential hypertension^75^^,^^76^^,^^78^ but do not have higher blood pressure^64^^,^^74^	association with hypertension is BMI dependent; deletion carrier individuals have lower diastolic blood pressure compared to BMI-matched copy-neutral individuals
Serum lipids	deletion carrier individuals have lower levels of HDL cholesterol and elevated triglycerides,^64^ putting them at increased risk for hyperlipidemia^78^	associations with lipid levels and hyperlipidemia risk are BMI dependent
Cardiovascular disorders	deletion carrier individuals are at increased risk for cardiac valve disorders and arrhythmias but not for ischemic heart disease^78^	associations with cardiac valve disorders and arrhythmias are BMI dependent
Hepatic function	ALT, AST, and GGT levels negatively correlate with CNV dosage,^64^ while ALP^64^^,^^200^ and albumin^64^ levels are increased and decreased among deletion carrier individuals, respectively; no increased risk for hepatic fibrosis among CNV carrier individuals,^78^ possibly due to underdiagnosis^207^	associations with ALT and albumin are BMI dependent; associations with AST, GGT, and ALP are partially independent of BMI
Gout	deletion carrier individuals have increased serum uric acid levels,^64^ as well as nominally significantly increased prevalence of gout^78^	association with serum urate is BMI dependent
Inflammation	deletion carrier individuals have increased CRP levels^64^^,^^75^^,^^77^	association with CRP is BMI dependent

Evidence linking features of the metabolic syndrome (other than adiposity) to 16p11.2 BP4-5 CNVs in the UK Biobank and the impact of conditioning these associations on body mass index (BMI)^28^ (Figure 2C). ALP, alkaline phosphatase; ALT, alanine aminotransferase; AST, aspartate aminotransferase; CRP, C-reactive protein; GGT, gamma-glutamyltransferase; HDL, high-density lipoprotein.

Epidemiologic data on the age of onset of metabolic phenotypes, as well as prevalence and efficacy of medication and lifestyle modifications, remain scarce. This is particularly relevant as other comorbidities could alter adherence to treatment strategies. Recently, long-term follow-up of two deletion carrier individuals treated with liraglutide (a glucagon-like peptide 1 analog) demonstrated effective weight loss accompanied by improved glycemia, lipidemia, and overall life quality.^208^ Offering promising perspectives, replication studies are required to establish the safety and efficacy of these therapies in deletion carrier individuals.

#### Reproduction

Dosage of 16p11.2 BP4-5 correlates with age at menarche in both clinical^25^ and population cohorts,^25^^,^^64^^,^^200^ with deletion and duplication carrier individuals experiencing menarche 1.5 years earlier and later than control subjects, respectively.^25^ As for other metabolic phenotypes, mouse models exhibit a reversed mirror effect on female sexual maturation, with duplication and deletion models experiencing earlier and delayed first ovulation, respectively.^25^ While childhood obesity causally lowers age at menarche,^209^ in humans the mirror effect was robust to correction for adult BMI.^25^ A similar effect is observed on relative age at first facial hair,^25^^,^^64^ suggesting that puberty timing is affected in both sexes. Conversely, age at menopause and balding are not altered.^64^ An Icelandic study found that deletion carrier individuals exhibited markedly reduced fecundity, while no effect was observed for the duplication carrier individuals.^70^ Males were more affected than females,^70^ an observation later generalized to a broader spectrum of rare deleterious mutations.^210^ Potential explanations include infertility, congenital malformations (see congenital anomalies of the genitourinary tract), or increased burden of neuropsychiatric disorders (see psychiatry and neurology) and other health outcomes that make it less likely to find a partner.^210^ In support of the former, sex hormone binding globulin levels, which regulate the amount of bioavailable testosterone, were reduced in UKBB deletion carrier individuals,^64^ even though this association was driven by increased BMI.^28^ Further research should disentangle contribution of these factors.

### Cardiac

Case reports have identified multiple congenital heart defects among 16p11.2 BP4-5 CNV carrier individuals.^32^^,^^39^^,^^211^^,^^212^^,^^213^^,^^214^^,^^215^^,^^216^^,^^217^^,^^218^^,^^219^^,^^220^ Within a study of 1,118 fetuses with congenital heart defects, 16p11.2 BP4-5 deletions were the second most common chromosomal alteration found in 0.9% of affected individuals.^221^ Penetrance of congenital heart defects among deletion carrier individuals is low, with estimates consistently ranging between 5% and 10%.^80^^,^^81^^,^^222^ Arguing in favor of a causal role of the region’s dosage, mouse models for the deletion present with subtle heterogeneous alterations in cardiac structure and function.^223^ Furthermore, more than 5% of BioVU CNV carrier individuals had cardiac findings in their electronic health records, with enrichment for “cardiac dysrhythmias” among deletion carrier individuals, and various cardiac congenital anomalies, cardiomegaly, and cardiac interventions (e.g., “heart transplant/surgery”) among duplication carrier individuals.^148^ Hence, congenital heart anomalies represent a rare but consequential feature of the 16p11.2 BP4-5 rearrangement with milder defects potentially contributing to cardiovascular diseases in adulthood (Table 2).

### Pulmonary

Thorough investigation of pulmonary phenotypes is lacking in clinical cohorts, despite isolated reports of early-onset asthma.^43^^,^^218^^,^^224^ In UKBB, deletion carrier individuals have reduced pulmonary function,^64^^,^^74^^,^^75^^,^^200^ as well as increased risk for asthma,^76^^,^^78^ chronic obstructive pulmonary disease (COPD),^78^^,^^200^ and respiratory failure.^75^ Similarly, BioVU CNV carrier individuals frequently presented with “abnormal findings during examination of lungs."^148^ While asthma risk was driven by an increase in BMI—a well-known risk factor for the disease—this was not the case for COPD and forced vital capacity, whose association was also independent of height.^28^ Recurrent pulmonary infections (see hematological and immune system) and environmental factors such as smoking, air pollution, and occupational or residential exposure to allergens, chemicals, dusts, fumes, or molds represent major risk factors for lung diseases. Except for tobacco smoking, whose rates are increased among UKBB CNV carrier individuals,^75^ very little is known about whether 16p11.2 BP4-5 CNV carrier individuals are differentially exposed to such factors and how these affect the expressivity of the rearrangement.

### Musculoskeletal and connective tissue

#### Global musculoskeletal features

16p11.2 BP4-5 CNV carrier individuals present with global musculoskeletal alterations. Shorter stature in deletion carrier individuals is reported in both clinical^81^ and population^64^^,^^74^^,^^75^^,^^200^ cohorts but only a fraction of population studies report taller stature in duplication carrier individuals.^74^^,^^200^ Adult levels of insulin-like growth factor 1 (IGF-1), which mediates the effect of growth hormone, are decreased in a BMI-dependent fashion among UKBB deletioncarrier individuals,^28^^,^^64^^,^^75^ possibly explaining the deletion carrier individuals’ short stature (Table 3). Bone composition is also affected, with the region’s dosage negatively correlating with heel bone mineral density.^64^^,^^74^^,^^200^ Even though obesity correlates with high bone mineral density^225^ the dosage effect is robust to BMI correction.^28^ Increased risk for arthrosis among UKBB deletion carrier individuals^78^ appears to be BMI driven,^28^ even though other mechanisms, such as structural anomalies of the joints, cannot be excluded. Indeed, joint hypermobility among clinically ascertained CNV carrier individuals has been described.^32^^,^^38^^,^^43^^,^^80^ Joint laxity—along with short stature, limb malalignment, and spinal deformity—is a hallmark feature of spondyloepimetaphyseal dysplasia with joint laxity type 2 (MIM: 603546) and autosomal-dominant disorder caused by mutations in the 16p11.2 gene *KIF22* (MIM: 603213) that often leads to early-onset arthrosis.^226^ Prevalence of spondyloepimetaphyseal dysplasia with joint laxity type 2 among deletion carrier individuals has not been assessed. Hinting at more global defects of connective tissues, there is emerging evidence linking the CNV to increased risk for various types of hernias (Table 3). UKBB CNV carrier individuals also exhibit decreased hand grip strength,^64^^,^^74^ paralleled by high rates of “muscle weakness” in BioVU deletion carrier individuals.^148^ While decreased muscle strength could not be explained by increased BMI and shorter stature,^28^ possible mechanisms include low IGF-1 levels, reduced physical activity, or neurological defects leading to hypotonia and muscle weakness.

**Table 3:** Emerging associations with 16p11.2 BP4-5 CNVs

**Phenotype**	**CNV**	**Context**	**Current evidence**	**Future directions**
**Neurology**				

Alzheimer disease	DUP	Alzheimer disease with psychosis shares disease mechanisms with SCZ^227^	2 DUP carrier individuals in 440 cases of severe Alzheimer disease with psychosis^228^	monitor older CNV carrier individuals for disease symptomatology, e.g., through longitudinal studies, and establish whether there is a parallel between neurodevelopmental and neurodegenerative diseases
Parkinson disease	DEL DUP	increased rate of tremors and dysrhythmia chiefly in DUP carrier individuals and reduced nimbleness of CNV carrier individuals in general^133^^,^^145^	case reports of a DEL carrier with PKD and dopa-responsive parkinsonism^188^ and a DUP carrier with levodopa-non-responsive early-onset parkinsonism^229^	
Hemiplegia	DEL	*PRRT2* mutations predispose to SeLIE, PKD, and ICCA and are more rarely leading to seizure and headache disorders^193^	DEL carrier individuals with benign nocturnal alternating hemiplegia of childhood^230^ and hemiplegic migraine^231^; frequent migraines in clinically ascertained DEL carrier individuals^30^ but no link in UKBB^78^	probe the link between *PRRT2* haploinsufficiency and disorders involving transient hemiplegia
Sleep disorders	DEL DUP	sleep apnea reports in DEL carrier individuals^43^^,^^80^^,^^224^^,^^232^; BMI-driven risk for sleep apnea in BioVU^148^ and UKBB^78^ DEL carrier individuals; mouse DEL model has altered sleep architecture (e.g., fragmented non-rapid eye movement sleep) and increased wake time^233^^,^^234^^,^^235^	sleep problems are common in CNV carrier individuals^30^: compared to familial controls, there is increased sleep disturbance and medical sleep concerns, but no difference in sleep duration^236^; no association with insomnia, hypersomnia, or narcolepsy in UKBB^78^	investigate sleep quality through objective approaches (e.g., polysomnography)
Spinal cord defects	DEL DUP	CNV carrier individuals are prone to spinal malformations	syringomyelia^80^^,^^81^^,^^158^^,^^237^ and spina bifida^80^^,^^81^^,^^232^^,^^237^^,^^238^^,^^239^^,^^240^ are reported, chiefly among DEL carrier individuals; increased risk for sciatica in UKBB DUP carrier individuals^76^	assess whether spinal cord defects are consequential to skeletal malformations

**Endocrinology & metabolism**				

Early insulin dysregulation	DEL	DEL carrier individuals are at increased risk for type 2 diabetes^75^^,^^76^^,^^200^	case reports of DEL carrier individuals with neonatal hyperinsulinemic hypoglycemia^241^^,^^242^ and hypoglycemic coma (fluctuating blood glucose)^224^	detailed glycemia/insulinemia assessment in pediatric cohorts to characterize the type, severity, and age of onset of insulin dysregulation
Insulin-like growth factor 1 (IGF-1)	DEL	DEL features, e.g., short stature and decreased muscle mass, could be explained by low IGF-1 levels	adult UKBB DEL carrier individuals have BMI-dependent decrease in IGF-1 levels^64^^,^^75^	establish onset of decreased IGF-1 levels and compare it to growth and weight gain trajectories
Type 1 diabetes	DEL	DEL carrier individuals are at increased risk for type 2 diabetes^75^^,^^76^^,^^200^	UKBB DEL carrier individuals have increased risk for type 1 diabetes^78^; the association is BMI dependent^28^	determine if the association results from early-onset type 2 diabetes cases misdiagnosed as type 1

**Connective tissue**				

Congenital diaphragmatic hernia (CDH)	DEL	CDH is a rare and life-threatening form of hernia^243^	3 DEL carrier individuals in 120 cases of CDH^244^^,^^245^; multiple other case reports^32^^,^^43^^,^^80^^,^^196^	stratify factors predisposing to different types of hernia, e.g., connective tissue weakness, pressure on abdominal organs due to spinal/thoracic deformities, cryptorchidism, or obesity and assess possible associations
Inguinal and umbilical hernia	DEL DUP	inguinal/umbilical hernias account for about 85% of repaired abdominal hernias^246^	case reports of inguinal/umbilical hernias in CNV carrier individuals^39^^,^^80^^,^^218^^,^^247^; conflicting evidence in UKBB^75^^,^^76^^,^^78^	

**Hematological**				

Neutrophils	DEL	DEL carrier individuals have lower immunity^78^^,^^200^^,^^248^ and decreased lymphocyte count^200^^,^^249^	neutrophil count is increased in UKBB DEL carrier individuals,^64^^,^^200^^,^^249^ despite reported cases of neutropenia^249^	
Platelets	DEL DUP	16p11.2 immunity gene *CORO1A* plays a role in platelet biology^108^^,^^109^	platelet count negatively correlates with CNV dosage in UKBB,^64^^,^^200^ whereas a thrombocytopenia case was reported in a DEL carrier^218^	probe the link between dosage and/or expression of *CORO1A* and platelet count
Reticulocytes	DEL DUP	DEL carrier individuals are at increased risk for (iron deficiency) anemia^76^^,^^78^^,^^15^	increased mean reticulocyte volume and decreased high light scatter reticulocyte count in UKBB DEL carrier individuals^200^; high fraction of immature reticulocyte in UKBB DUP carrier individuals^200^	better characterize changes in reticulocytes and relate them to anemia risk

**Sensory organs**				

Audition	DEL DUP	sensory processing is affected in CNV carrier individuals^88^^,^^89^^,^^90^ with atypical neural activity upon auditory stimuli^170^^,^^171^^,^^172^	auditory dysfunction in 9.5% of DEL^32^^,^^39^^,^^43^^,^^81^ and 3.7% of DUP^80^ carrier individuals, respectively	systematically characterize auditory dysfunctions and analyze hearing tests in UKBB and/or clinical cohorts
Ophthalmic findings	DEL DUP	sensory processing is affected in CNV carrier individuals^88^^,^^89^^,^^90^ with atypical neural activity upon visual stimuli^173^^,^^174^	frequent ocular findings^30^^,^^159^: strabismus and refractive errors in >5% of CNV carrier individuals^30^^,^^32^^,^^80^^,^^212^; abnormal eye convergence in 11% of DEL and 30% of DUP carrier individuals, respectively^133^; major anomalies or blindness in 2.6% of DEL and 1.5% of DUP carrier individuals, respectively^80^; no link to cataract, glaucoma, and cornea disorders in UKBB^78^	comprehensive ophthalmologic examination and analyze refractometer, intraocular pressure, and visual acuity measurements in UKBB and/or clinical cohorts

**Cancer**				

Neuroblastoma	DEL	the region harbors genes in the MAPK/ERK pathway, linked to cancer; tumors and cancers have rarely been reported among CNV carrier individuals^80^^,^^250^	22 DEL carrier individuals in 5,585 neuroblastoma cases, all lacking concurrent *MYC* (MIM: 190080) amplification (i.e., less aggressive neuroblastoma form)^251^	assess molecular mechanisms linking the DEL to neuroblastoma specifically and tumorigenesis more broadly

Phenotypes recently linked to 16p11.2 BP4-5 CNVs but whose associations await confirmation. Phenotypes are ordered by physiological category and the main implicated CNV type is indicated. Context provides complementary information to support or nuance the currently available evidence. Future directions that could help to confirm, refine, or refute the associations are given. CNV/DEL/DUP refer to 16p11.2 BP4-5 rearrangements. CNV, copy-number variant; DEL, deletion; DUP, duplication; BioVU, Vanderbilt University Medical Center’s biobank; BMI, body mass index; ICCA, infantile convulsion with choreoathetosis syndrome; PKD, paroxysmal kinesigenic dyskinesia; SCZ, schizophrenia; SeLIE, self-limited familial and non-familial infantile epilepsy; UKBB, UK Biobank.

#### Craniofacial features

The mirror effect on head circumference—making deletion and duplication carrier individuals more prone to macro- and microcephaly, respectively—represents one of the first described hallmarks of the 16p11.2 BP4-5 rearrangement^41^^,^^43^^,^^80^^,^^81^^,^^133^ and was later paralleled by changes in brain volume (see structural & functional alterations of the nervous system). Head circumference correlates with BMI and a third of obese deletion carrier individuals are macrocephalic.^80^^,^^81^ Mechanistically, modulating expression of the 16p11.2 *KCTD13* gene recapitulates the head size phenotype through perturbation of RhoA signaling.^23^^,^^26^^,^^252^^,^^253^ In zebrafish, *kctd13* expression negatively correlates with proliferation of neuronal progenitor and overexpression of the human ortholog increases apoptosis.^23^ While modulating *kctd13* expression is sufficient to establish the neuroanatomical changes, expressivity is increased by simultaneously altering expression of two other 16p11.2, *MVP* and *MAPK3*^23^, suggesting *cis*-epistatic **genetic interactions**^23^ (Box 1). Concordantly, dysregulation of the ERK signaling cascade—of which MAPK3 is part—was suggested to play a role in increasing progenitor proliferation and decreasing hippocampal synaptic protein synthesis in a mouse deletion model.^254^^,^^255^ Increased dendritic arborization in a duplication mouse model was linked to the same kinase cascade.^256^ Another study investigating global craniofacial features found that individual overexpression of seven 16p11.2 BP4-5 genes in zebrafish induced an analogous phenotype to the lower jaw protrusion observed in human duplication carrier individuals.^18^ Simultaneous overexpression of human *KCTD13*, *MAPK3*, and *MVP* yielded an even stronger phenotype, even though none of these genes showed an effect individually.^18^ Additionally, in humans, mild positive and negative dosage effects on nasal and frontal regions, respectively, were identified from 3D morphometric imaging.^18^ These align with frequently reported facial features—broad forehead, micrognathias, or flattened profile—despite no recognizable facial gestalt.^32^^,^^38^^,^^43^^,^^81^^,^^218^ Skull deformities, such as **craniosynostosis** (Box 4), are present in 1.3% of deletion carrier individuals^39^^,^^80^^,^^81^^,^^238^ and can lead to Chiari type 1 malformation (see structural and functional alterations of the nervous system). Rarely, more severe malformations of the **posterior fossa** (Box 4) have been reported.^257^^,^^258^ Overall, 16p11.2 BP4-5 dosage negatively correlates with head circumference and predisposes to mild dysmorphic features and cranial anomalies. These have low penetrance, especially among duplication carrier individuals and non-medically ascertained deletion carrier individuals.^81^

#### Spine and thoracic cage deformities

Deformities of the spine and thoracic cage are recurrent among 16p11.2 BP4-5 deletion carrier individuals.^38^^,^^43^^,^^80^^,^^81^^,^^212^^,^^247^^,^^259^^,^^260^ Individuals carrying the deletion or a loss-of-function variant in the 16p11.2 gene *TBX6* (MIM: 602427) in combination with a **hypomorphic** (Box 1) *TBX6* allele explain up to 11% of congenital scoliosis cases in a Chinese population.^237^ Highlighting *TBX6* as the causal gene for spinal malformations, these results were since replicated.^239^^,^^261^ Further research showed that *TBX6*-associated congenital scoliosis has distinguishable endophenotypes including earlier onset, increased prevalence of hemivertebrae and rib anomalies, and lower rates of spinal cord defects.^262^ *TBX6* compound inheritance also associates with a broad spectrum of disorders of vertebral development and segmentation—ranging in severity from scoliosis (abnormal sideways curvature of the spine) or kyphosis (abnormal forward rounding of the spine) to generalized defects such as **spondylocostal dysostosis** (Box 4) (MIM: 122600),^214^ as well as a cooccurrence of structural defects of the vertebra, ribs, and kidney^263^ (see congenital anomalies of the genitourinary tract). Effects of increased dosage of *TBX6* are less well defined, although duplication carrier individuals have been reported to suffer from congenital vertebral malformations,^43^^,^^196^^,^^264^ which tends to affect the upper spine (i.e., cervical vertebra),^264^ in contrast with the higher predisposition to lower spine defects (i.e., thoracic and lumbar vertebra) in deletion carrier individuals.^237^^,^^262^ Note that *KIF22*-associated spondyloepimetaphyseal dysplasia with joint laxity type 2 is also characterized by spinal deformities,^226^ so that additive or epistatic interactions between 16p11.2 genes could contribute to heterogeneity in skeletal phenotypes. While we did not identify an association with scoliosis in UKBB (data not shown), BioVu deletion carrier individuals had higher diagnostic rates of “congenital musculoskeletal deformities of the spine.”^148^

### Genitourinary

#### Congenital anomalies of the genitourinary tract

About 6.3% of European^265^ and 0.9%–1.4% Chinese^266^^,^^267^ females with **Müllerian aplasia** (Box 4) (MIM: 277000; 601076) carry the 16p11.2 BP4-5 deletion. Similarly, haploinsufficiency of *TBX6*—through deletion or point mutations—was identified in 23 out 112 individuals with Müllerian aplasia,^268^ as well as in one individual with distal vaginal atresia but normally developed uterus and cervix.^269^ These findings parallel the increased rate of female reproductive tract disorders observed among Estonian Biobank CNV carrier individuals, which have been proposed to be driven by dosage of *ASPHD1* and *KCTD13* based on Mendelian randomization and single-gene dosage modulation in zebrafish.^25^ Anomalies of the male genitalia, including cryptorchidism (undescended testis), hypospadias (mislocalization of the urethra’s opening), and micropenis, have also been reported in 16p11.2 BP4-5 deletion^38^^,^^43^^,^^196^ and less frequently in duplication^43^^,^^44^ carrier individuals. After identifying an enrichment of 16p11.2 BP4-5-overlapping deletions among individuals with genitourinary defects, mouse studies showed that decreased dosage of *Kctd13* associated with penile and testicular anomalies,^270^ while reduced dosage of *Maz* led to defects of the upper genitourinary tract and high penetrance of congenital anomalies of the kidney and urinary tract (CAKUT).^271^ CAKUT describes a broad spectrum of phenotypes—including kidney anomalies, ectopic or horseshoe kidneys, obstructive uropathies, and **vesicoureteral reflux** (Box 4)—and was reported in a small fraction of early descriptive studies of 16p11.2 BP4-5 CNV carrier individuals.^32^^,^^43^ Echoing the finding that 0.5% of fetuses with ultrasound renal anomalies harbored a 16p11.2 BP4-5 deletion,^272^ the deletion was enriched in a cohort of 2,800 CAKUT-affected individuals.^58^ Unlike other recurrent CNVs, 16p11.2 BP4-5 deletions are linked to a broad spectrum of genitourinary defects.^58^^,^^273^ Using a genotype-first approach, another study found that 13 of 52 deletion carrier individuals presented with defects of the urinary tract,^274^ establishing the deletion as an important risk factor for CAKUT. Frequent co-occurrence of skeletal and genitourinary malformations^263^ has led to the hypothesis that haploinsufficiency of *TBX6* is at the origin of both phenotypes. Concordantly, mouse models with reduced *Tbx6* expression exhibit CAKUT phenotypes.^58^^,^^274^ This implicates *TBX6* dosage as the driver of both skeletal and genitourinary phenotypes, with dosage of *cis*-genes (i.e., *KIF22*, *KCTD13*, *MAZ*, and *ASPHD1*) likely contributing to phenotypic variability.

#### Renal function

16p11.2 BP4-5 deletion carrier individuals were enriched in a cohort of 6,679 chronic kidney disease cases.^275^ Accordingly, UKBB CNV, and chiefly deletion carrier individuals, have increased levels of the renal biomarker cystatin C^64^^,^^75^^,^^77^ and were at increased risk for both chronic kidney disease (CKD) and acute kidney injury (AKI).^76^^,^^78^^,^^200^ The region’s dosage positively correlated with serum creatinine levels.^64^^,^^200^ Impaired kidney function is typically associated with increased creatinine levels, suggesting that muscle wasting or liver diseases, which are observed in deletion carrier individuals, could play a role in the lowered creatinine levels. Importantly, the U-shape effect on cystatin C and AKI and the mirror effect on creatinine were robust to BMI adjustment^28^ (Figure 2C; Table 2), putting forward the hypothesis that mechanisms other than obesity, such as subclinical structural renal alterations, affect renal function in the long term.

### Hematological and immune system

16p11.2 BP4-5 deletion carrier individuals are at increased risk for anemia, and in particular iron deficiency anemia.^15^^,^^76^^,^^78^ Anemia risk was associated with the number of copies of *BOLA2*, a gene involved in cellular iron homeostasis mapping within the 16p11.2 BP4-5 flanking breakpoints (Figure 1A) and present in 3–8 copies in humans.^4^^,^^15^ These *Homo sapiens*-specific copy-number polymorphic duplications of *BOLA2* are under positive selection and were suggested to provide an adaptive role in protecting against iron deficiency.^4^^,^^15^

There is also evidence that the myeloid and lymphoid lineages are compromised in deletion carrier individuals. Severe combined immunodeficiency 8 with T lymphocytopenia (MIM: 615401) has been reported in deletion carrier individuals **compounded** (Box 1) with mutations in the 16p11.2 T cell-mediated immunity and platelet biology gene *CORO1A*^108^^,^^276^ (Table 3), while immune deficiency was suspected in three independent deletion carrier individuals with severe pneumonia or low immunoglobulins.^39^^,^^218^^,^^224^ A retrospective analysis of 170 deletion carrier individuals ascertained for ASD revealed that 81% had a history of significant infection, including recurrent otitis (28%), chronic bronchitis (4%), or pneumonia (26%).^248^ Low lymphocyte levels have been reported in UKBB^200^^,^^249^ deletion carrier individuals along with increased risk for pneumonia,^78^^,^^200^ which are not secondary to increased adiposity.^28^ Current evidence indicates that the 16p11.2 BP4-5 deletion represents a risk factor for both anemia and recurrent infections.

### Emerging findings

The pleiotropy of recurrent CNVs is likely to be currently underestimated.^78^ Accordingly, there is emerging evidence for a link between 16p11.2 BP4-5 CNVs and several additional phenotypes (Table 3). Future studies will be required to probe the robustness of these associations by estimating the prevalence of the CNV in cohorts ascertained for these phenotypes and through specialized follow-up analyses.

## Embrace diversity to better understand phenotypic heterogeneity

**Figure 3:**
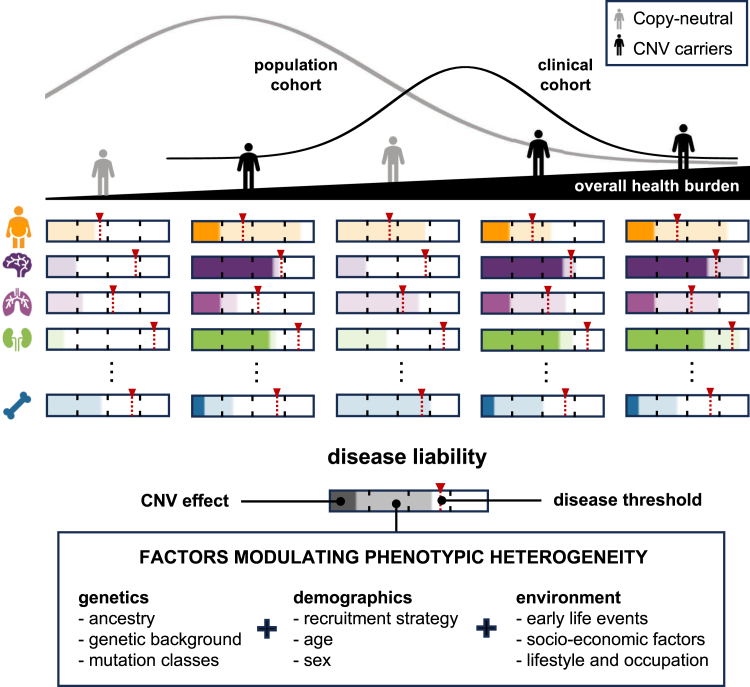
Model of phenotypic variability among CNV carrier individuals Schematic view on a holistic approach to understanding phenotypic heterogeneity. Top: distribution of the global health burden among copy-neutral (gray) and CNV carrier (black) individuals. CNV carrier individuals from population cohorts tend to be sampled from the left side of the CNV carrier distribution, while CNV carrier individuals from clinical cohorts tend to be sampled from the right side of that distribution. Bottom: liability to diseases affecting different physiological systems for five individuals sampled from the above distributions. The red mark represents the liability threshold that needs to be exceeded for an individual to be diagnosed with a disease. The threshold is lower for common diseases and individuals that are near the threshold might present with subclinical features, e.g., the first individual is overweight without meeting diagnostic criteria for obesity (orange). The dark-colored area represents the contribution of the CNV to disease liability, which in the absence of epistasis or gene-environment interactions is constant across CNV carrier individuals but variable across diseases. Typically, contribution is stronger for rare disorders, but usually not sufficient to pass the threshold. The light-colored area represents the contribution of various other factors to disease liability, which will determine whether the individual reaches the disease threshold or not. Importantly, contribution of these factors is variable across both diseases and individuals, resulting in phenotypic heterogeneity across CNV carrier individuals.

Over the years, numerous studies have demonstrated the extensive pleiotropy of 16p11.2 BP4-5 CNVs, establishing the rearrangement as an important susceptibility locus for a wide range of disorders. As such, diagnostic finding of the CNV is typically disclosed to affected individuals. Yet, in 90,595 participants of the Geisinger MyCode Community Health Initiative health system, less than 10% of carrier individuals of a CNV associated with a genomic disorder had received a clinical diagnosis, despite exhibiting clinical features associated with the condition.^65^ From a personalized medicine perspective, this emphasizes the importance of adopting a holistic approach, allowing diagnosis of individuals with milder and/or atypical presentation, as well as follow-up by multiple specialists to anticipate and potentially treat and/or prevent future complications. This is particularly relevant, given the highly heterogeneous clinical manifestation of 16p11.2 BP4-5 CNVs (Figure 3). While it is rare to observe all associated phenotypes within a single individual, further research is required to determine which constellation of symptoms is more likely to co-occur and how the latter is impacted by ascertainment. To achieve this, it is imperative to (1) define and bring awareness to clinicians of the spectrum of possible manifestations of the rearrangement, (2) understand factors contributing to phenotypic heterogeneity, and (3) gain mechanistic insights into the molecular pathways connecting altered gene dosage to phenotype. Here, we discuss some key areas that might allow filling missing knowledge gaps, while emphasizing how diversity in affected individuals, experimental models, and analytical approaches can catalyze discoveries.

### Diversity in ascertainment and demographics

Results from clinical and population studies often converge on similar physiological systems. Yet, both types of studies suffer from ascertainment biases, leading to over- and under-estimation of the CNV’s effect, respectively, and exposing the two extremities of the same phenotypic continuum. The latter ranges from subtle subclinical alterations—as often seen in transmitting parents of clinically ascertained carrier individuals identified by cascade testing^277^^,^^278^ or carrier individuals from population cohorts—to severe medical conditions observed in probands from clinical cohorts. Hence, results of clinical and population cohorts should be seen as complementary, investigating the same question but from a different angle.

Differences in ascertainment mean that clinical and population cohorts have different demographics. Clinical cohorts tend to be enriched for severe pediatric cases, while population cohorts are usually composed of adults with longitudinal follow-up data. This offers the opportunity to investigate age at disease onset and clinical trajectory, especially for phenotypes expressed only later in life, which are often less well characterized among carrier individuals of syndromic CNVs. Indeed, not only do 16p11.2 BP4-5 CNV carrier individuals suffer from an increased risk for a broad range of common diseases, but they also suffer from an earlier age at onset compared to individuals with a different disease etiology.^78^ This information can be tapped to establish preventive measures that anticipate and attenuate later-onset comorbidities.

Another important consideration relates to sex. Clinical cohorts are typically recruited with a phenotype-first approach but many traits exhibited skewed male-female ratios, impacting sex representation and leading to biases in the clinical description of comorbidities. For instance, ASD—a hallmark feature of the 16p11.2 BP4-5 rearrangement—has an estimated male-to-female ratio of 3:1.^279^ While true differences in disease prevalence, behavioral symptoms, and neurobiological profiles between sexes exist,^280^ underdiagnosing of ASD among females due to differences in clinical presentation and/or societal stereotypes is probably widespread.^279^^,^^281^ Furthermore, factors such as comorbidities and genetic etiology impact sex ratio estimates.^282^ The sex ratio across 16p11.2 BP4-5 deletion carrier individuals appears stable—about 1.5 male per female carrier individual—across different ascertainment strategies (Table 4). For the duplication, there are about twice as many male carrier individuals in clinically ascertained cohorts, compared to an almost equal sex ratio in population cohorts (Table 4). Unlike what would be expected from a female-protective effect,^283^ UKBB is significantly depleted of female deletion carrier individuals (Table 4). One explanation could be sex-specific differences in participation compounded over multiple traits affected by the deletion, as suggested by the widespread genetic correlation between sex and adipose or psychiatric traits.^284^ This would mean that females with hallmark features of the deletion, such as increased BMI and decreased cognitive ability, are less likely to participate. In line with this, the BMI-increasing *FTO* (MIM: 610966) allele has a higher frequency in male UKBB participants,^284^ suggesting that obese females are less likely to enroll in biobanks. Differences in prevalence across sexes might reflect genetic interaction with sex but little is known about single-sex or sex differential effects of 16p11.2 BP4-5 CNVs. Rodent deletion models show broad sex- and age-specific behavioral differences,^17^^,^^285^ as well as male-specific sleep,^233^ reward-learning,^286^ neurovascularization,^287^ and vocal communication^288^ impairments, while females exhibit increased levels of anxiety.^289^ Similarly, social behavior and reaction to novel object are more affected in male rat models.^17^ In humans, a significantly stronger reduction in fecundity was observed in male deletion carrier individuals,^70^ while another study found that female CNV carrier individuals ascertained for developmental delay and intellectual disability experienced a larger number of comorbidities.^282^ In the future, potential sex differences for each of the broad range of traits associated with 16p11.2 BP4-5 rearrangements should be investigated.

**Table 4:** Sex ratio among 16p11.2 BP4-5 CNV carrier individuals with different ascertainments

**Ascertainment**	**CNV status**	**Male (%)**	**Female (%)**	**Ratio**	***p***
Autism spectrum disorder^282^	deletion	9 (56%)	7 (44%)	1.3:1	0.061
	duplication	6 (67%)	3 (33%)	2.0:1	0.420
	cohort	4,588 (78%)	1,284 (22%)	3.6:1	
Developmental delay and intellectual disability^282^	deletion	45 (61%)	29 (39%)	1.6:1	0.910
	duplication	29 (64%)	16 (36%)	1.8:1	0.547
	cohort	17,061 (60%)	11,492 (40%)	1.5:1	
Population cohort (UKBB)^64^^,^^78^	deletion	45 (62%)	28 (38%)	1.6:1	0.009^∗^
	duplication	41 (46%)	48 (54%)	0.9:1	1
	cohort	152,967 (46%)	178,555 (54%)	0.9:1	

Number and percentage of male and female 16p11.2 BP4-5 deletion and duplication carrier individuals for two clinical cohorts ascertained for autism spectrum disorder or developmental delay/intellectual disability, and the UK Biobank (UKBB). Sex distribution of each cohort is indicated as a third row. Sex ratio indicates the number of males per female in the considered sample. *p* values of two-sided Fisher tests are reported, assessing differences in sex ratio among CNV carrier individuals, compared to the entire cohort. Significant result (*p* ≤ 0.05) is indicated with an asterisk.

### Diversity in genetic background

There is a significant correlation between a CNV carrier individual’s cognitive and social skills and those of non-carrier first-degree relatives, indicating that “familial background” modulates phenotypic expressivity, with similar effects in deletion and duplication carrier individuals.^81^^,^^136^^,^^137^ The latter encompasses many genetic variants that can be grouped depending on their frequency (rare versus common) and phenotypic impact (large versus small).

Early studies hypothesized that additional rare variants sensitize genomes, leading to differential phenotypic expression of the same 16p11.2 BP4-5 rearrangement.^290^ Validating this “two-hit” theory, a large fraction of 16p11.2 BP4-5 duplication (16%) and deletion (8%) carrier individuals were found to harbor a second large CNV and these individuals exhibited a more severe and diverse phenotype.^33^ A later study found that 70% of clinically ascertained 16p11.2 BP4-5 CNV carrier individuals harbored a rare secondary CNV and that there was a strong maternal transmission bias for pathogenic secondary deletions.^31^ Similarly, the number of secondary rare and predicted-to-be pathogenic variants negatively correlated with cognitive function and head circumference in 16p11.2 BP4-5 deletion carrier individuals.^277^ Sometimes, the second hit is linked to known genetic disorders, such as severe combined immunodeficiency,^276^ Cohen syndrome (MIM: 216550),^291^ Mowat-Wilson syndrome (MIM: 235730),^292^ Zellweger spectrum disorders (MIM: 614862),^241^ or Friedreich ataxia (MIM: 229300),^293^ leading to more severe cases with atypical presentation and highlighting dual diagnosis as an explanation for phenotypic heterogeneity.^294^ While syndrome coexistence or bi-parental inheritance^9^^,^^10^ might occur at random, the phenomenon might be fostered by cross-disorder assortative mating.^278^ Future work aiming at characterizing the interaction between assortative mating, CNV inheritance mode, and parent-of-origin effects is required to determine how phenotype severity and heterogeneity is compounded over generations.

There is now also emerging evidence that genome-wide **polygenic scores (PGSs)** (Box 1) act additively to CNVs. For instance, 16p11.2 BP4-5 duplication carrier individuals with a PGS predisposing to high BMI tend to exhibit a less severe reduction in BMI than those with a PGS predisposing to low BMI, with opposite trends in deletion carrier individuals.^295^ Another study showed that SCZ-affected individuals carrying an SCZ-associated CNV had lower SCZ PGS than those that did not.^296^ Because (1) PGS reduction was inversely proportional to the CNV’s effect on SCZ and (2) the 16p11.2 BP4-5 duplication substantially contributes to SCZ risk, SCZ PGS was not a significant predictor among duplication carrier individuals.^296^ These studies suggest that CNV expression is modulated by multiple common variants with minute effects, extending the “two-hit” model to a polygenic one. Studying the contribution of the polygenic background is complicated by healthy volunteer bias and assortative mating, highlighting the need for further research to understand how different mutations act in concert to determine an individual’s genetic liability for a given trait.

### Diversity in ancestry

An important source of diversity stems from genetic ancestry. A key question is whether the frequency of the 16p11.2 BP4-5 rearrangement varies across ancestries. Deleterious CNVs are less prevalent in UKBB individuals of non-European ancestry.^123^ This could be explained by some haplotypes, e.g., at cytobands 17q21.31 and 16p12.1, favoring genetic rearrangements due to the size and/or orientation of encompassed segmental duplication blocks, making some populations more susceptible to *de novo* CNVs.^290^ This does not seem to be the case for 16p11.2 BP4-5,^4^ despite archaic introgression in this cytoband in some populations.^297^ Accordingly, neither the ASD Simons Foundation Powering Autism Research for Knowledge cohort (SPARK; *N* = 58,419; 20% non-European)^123^ nor the healthcare cohort Bio*Me* (*N* = 24,877; 68% non-European)^66^ identified a significant divergence in 16p11.2 BP4-5 CNV prevalence across ancestries, even though estimates are limited by the relatively small sample size of each ancestry group. Alternatively, differences in the frequency of other mutations might modulate CNV expressivity, making certain phenotypes more common in specific populations. For instance, autosomal-recessive phenotypes might be more frequent in carrier individuals from a population in which loss-of-function alleles are widespread, as illustrated by the high prevalence of congenital scoliosis in deletion carrier individuals of Asian ancestry due to the high frequency (44%) of a *TBX6* hypomorphic haplotype, which is rarer in individuals of European (33%) and African (<1%) ancestries.^237^ Similarly, compounding the deletion with a haplotype associated with reduced MAPK3 expression affects early neuronal development.^298^ Conversely, one could expect phenotypes to become apparent in populations of duplication carrier individuals in which relevant **hypermorphic** (Box 1) alleles are widespread, even though, to our knowledge, no such example has been reported for the 16p11.2 BP4-5 CNVs. A major limitation is that except for a few large studies in individuals of Asian ancestry, the bulk of current knowledge stems from investigating CNV carrier individuals of European ancestry (Box 3).

### Diversity in environment

Environmental exposures represent potent factors that contribute to phenotypic heterogeneity, but their role remains unexplored. One study found that an increased number of perinatal events (e.g., preterm birth, abnormal presentation, low birthweight, or respiratory distress), but not prenatal events, led to increased ASD symptomatology among deletion carrier individuals.^299^ In duplication mouse models, adolescent exposure to the psychoactive constituent of cannabis exacerbated deficits in social memory in adulthood,^300^ while *Mapk3* knockout mice are hypersensitive to the rewarding properties of morphine.^165^ Many other exposures during childhood, adolescence, and adulthood, including diet, smoking habitat, alcohol consumption, physical activity, sleep hygiene, medication usage, exposure to pollutants, occupation, socio-economic status, and access to medical care, could impact penetrance, expressivity, and age of onset of diseases associated with the rearrangement. Future studies should assess whether environmental factors exacerbate (or mitigate) clinical features beyond simple additive effects, i.e., through CNV-environment interactions. For common variants, it was demonstrated that genetic effects are modulated by different environments between populations, more so than by true differences in causal effects across ancestries.^301^ Hence, it would be useful to identify environmental exposures that prompt more severe expression of certain phenotypes, as well as factors such as early genetic diagnosis or follow-up by a multidisciplinary team, that have the potential to alleviate the symptomatologic burden.

### Diversity in mutation classes

A long-standing challenge relates to linking genetic content to specific phenotypic features. Besides experimental approaches (see diversity in experimental approaches), existing genetic diversity at the locus can be leveraged to gain functional insights. Larger rearrangements, e.g., between BP1 and BP5 (Figure 1A), demonstrated the additive contribution of the proximal BP4-5 and the distal BP2-3 regions to BMI and head circumference,^302^ while a smaller 118 kb deletion encompassing only *MVP*, *CDIPT* (MIM: 605893), *SEZ6L2*, *ASPHD1*, and *KCTD13* was found to segregate with ASD features over three generations.^303^ Due to the absence of segmental duplications between BP4 and BP5, reports of partial rearrangements are sparse. Alternatively, rare protein-coding variants can provide insights into gene functionality, as exemplified for *PRRT2* and *TBX6*, the genes associated with PKD^304^ and scoliosis,^237^ respectively. While these examples have been elucidated through family studies, alternative approaches exist for population cohorts such as **burden tests** (Box 1) or more elaborated variance component and combination tests (e.g., SKAT-O^11^). These have been performed in the UKBB for a wide spectrum of traits^305^^,^^306^ but did not yield any significant association for 16p11.2 BP4-5 genes, except for an association between *SLX1A* and cannabis usage.^306^ The limitation of these tests is that rare variants account for only a small fraction of **heritability** (Box 1), which is concentrated on a few, highly constrained genes.^307^

**Figure 4:**
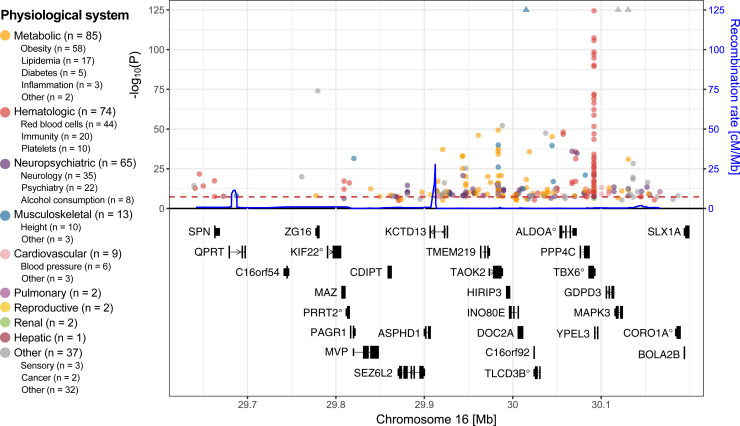
GWAS Catalog associations at 16p11.2 BP4-5 Top: 290 single-nucleotide variants associations mapping to the 16p11.2 BP4-5 CNV region (GRCh38) reported in the GWAS Catalog^309^ (accessed March 14^th^, 2024). The negative logarithm of the association *p* value (P; left y axis) is plotted against the genomic position (x axis). The dashed red line represents the threshold for genome-wide significance, *p* < 5 × 10^−^^8^. Associations are plotted from the suggestive *p* value of *p* < 7 × 10^−^^6^. *p* values for three signals, depicted as upward-facing triangles, were truncated. Associations are colored according to physiological systems. Number of signals for each category and subcategory is reported (*n*). The GRCh38 recombination rate in cM/Mb is depicted in blue (right left axis) and was downloaded from the Eagle software^311^ webpage. Bottom: exonic structure of protein-coding genes overlapping the region. ° indicates Online Mendelian Inheritance in Man (OMIM) morbid genes.

Common variants account for a much larger fraction of trait heritability and can also be leveraged to increase confidence in the causal role of the locus. For instance, early GWASs found the BP4-5 variant rs4583255[T] to increase risk for psychosis while decreasing BMI, mimicking two hallmarks of the duplication.^308^ To date, 290 associations with single nucleotide variants have been mapped to the region and reported in the NHGRI-EBI GWAS Catalog^309^ (Figure 4). Paralleling observations in CNV carrier individuals, most signals relate to metabolic, hematologic/immune, and neuropsychiatric phenotypes. Associations with other traits linked to 16p11.2 BP4-5 CNVs more recently, such as platelet count or diabetes, are also reported. This supports CNV findings through independent genetic perturbations converging onto the same phenotypic changes, although the significance of the observed trait overlap has not been rigorously assessed via statistical tests. One caveat is that the lack of recombination prevents accurate mapping of these signals to specific causal genes. Strategies to contend with this include incorporation of molecular data such as transcriptomics or proteomics. For instance, variants associated with gene expression—commonly known as expression quantitative loci (eQTLs)—were used to estimate the impact of changes in expression of 16p11.2 BP4-5 genes on hematological traits using **Mendelian randomization**^249^ (Box 1). The approach identified decreased expression of *CORO1A*, *KIF22*, and *BOLA2-SMG1P6* as causally decreasing lymphocyte count, thereby mimicking both the decreased gene expression expected from the region’s deletion and the decreased lymphocyte count observed in deletion carrier individuals.^249^ Few studies have successfully incorporated other mutation classes to gain functional insights, but this strategy has not been systematically explored. Of course, this approach assumes that a single or maybe a few genes in the region are causal for a given phenotype.^310^ While this model might be true for some phenotypes, others might have a polygenic basis, possibly involving interactions with genes in and beyond 16p11.2 BP4-5.^5^^,^^22^^,^^26^^,^^111^^,^^112^^,^^302^

Pairwise gene knockdown experiments (Box 2) revealed intraregional epistatic interactions. Interestingly, a mouse model hemi-deleted for three genes (*Taok2*, *Sez6l2*, and *Mvp*) recapitulates behavioral alterations observed in 16p11.2^*Del/+*^ mice, while the additional hemi-deletion of *Mapk3* decreased phenotypic similarities.^27^ However, another mouse model hemi-deleted for *Mapk3* and *Mvp* leads to altered behavioral performances.^20^ This suggests that phenotypes linked to the CNVs can be recapitulated through perturbation of various gene combinations, implying redundancy. Supporting the contribution of multiple genes to the same phenotype, a study found that phenotypic variance was better explained by pairwise, as opposed to single, human gene expression.^312^ Combinatorial knockdown and overexpression experiments, as well as transcriptome-wide studies of gene expression dysregulation induced by the rearrangement also revealed widespread interactions between 16p11.2 BP4-5 orthologs and other developmental delay and intellectual disability genes, genomic disorder regions, and ciliopathy genes.^5^^,^^22^ For instance, long-range interactions between BP4-5 and BP2-3 are evolutionarily conserved^22^ (Figure 1A). These two rearrangements generate similar phenotypes in humans, including increased risk for ASD and a mirror effect on BMI and head circumference.^112^ Specifically, mouse and zebrafish orthologs of the BP2-3 gene *LAT* (MIM: 602354) act in concert with *KCTD13*—the major BP4-5 driver of head circumference^23^—to modulate brain size, with additional contributions of *MVP* and *MAPK3*.^26^^,^^302^ Chromatin conformation assays further demonstrated high levels of interaction between the two regions,^112^^,^^313^ as well as the entire short arm of chromosome 16 (16p), which was found to harbor the greatest excess of ASD common polygenic influence.^111^ This suggests that CNVs in the region lead to broad disruption of local 3D genomic structure, possibly explaining why human cortical organoids derived from deletion carrier individuals induce global downregulation of neuronally expressed 16p genes,^111^ including genes linked to developmental delay, intellectual disability, and psychiatric conditions, such as the splicing regulator *RBFOX1* (MIM: 605104).^314^ Intriguingly, increased local 16p PGSs for ASD exerted a similar impact on gene expression,^111^ reconciling the rare and common component of an individual’s familial background.

### Diversity in experimental approaches

The establishment of the core features of 16p11.2 BP4-5 CNV carrier individuals prompted the study of the region in controlled experimental settings through animal and human cellular models (e.g., induced pluripotent stem cells) to gain mechanistic insights into the molecular pathways that connect altered dosage to disease features. They can be broadly divided into models that study the impact of the entire CNV versus those that independently assess the function of each of the genes mapping to the interval, sometimes using combinatorial approaches (Box 2). By controlling environmental variables and allowing engineering of precise genetic alterations, model organisms allow dissection of the individual contribution of the different genes at the locus. These experiments catalyzed the development of pharmacological interventions—often targeting the GABAergic^16^^,^^315^^,^^316^ and serotonin^317^^,^^318^^,^^319^^,^^320^ systems—that improve cognitive and behavioral responses in mouse models of the CNV. Similarly, inhibition of RhoA signaling partially restored neuronal morphology and migration, as well as functional and cognitive deficits in cellular and mouse models,^153^^,^^154^^,^^321^ while inhibiting the ERK pathway rescued anatomical and behavioral deficits in cellular models of the duplication^256^ and a mouse model of the deletion,^322^ respectively. Recently, phospho-proteomics revealed dysregulation of mTOR signaling as a common mechanism leading to neural precursor cell defects in both 16p11.2 BP4-5 deletion carrier individuals and idiopathic ASD-affected individuals,^323^ highlighting how new molecular insights can be gained through multi-omics studies. Yet, because all models present their own limitations, it is important to replicate results across multiple experimental strategies and validate findings in humans to ensure their robustness and clinical utility.^324^ Indeed, a recent study performing transcriptional and functional profiling across various mouse tissues and human-derived cellular models emphasized the strong context dependency of transcriptomic, morphological, electrophysiological, and cell-fate signatures of 16p11.2 CNV models.^6^

## Conclusions

The 16p11.2 BP4-5 rearrangement represents one of the most common etiologies of genomic disorders, leading to a broad and variable spectrum of phenotypes that extends far beyond neurodevelopmental disorders. Poor awareness around the syndrome and heterogeneous phenotypes that require personalized solutions have been described as a challenge to parents of children affected by 16p11.2 BP4-5 CNVs in accessing adequate and continued support.^325^ To ensure equity in diagnosis and provide personalized treatment plans, physicians must be aware of the different clinical presentations of these CNVs and assemble multidisciplinary teams of specialists who can anticipate and manage the different associated comorbidities.^87^^,^^326^ This task is complicated by our lack of understanding of the specific genetic and environmental factors that contribute to phenotypic heterogeneity. Yet, surveys of both parents of pediatric 16p11.2 BP4-5 deletion carrier individuals and adults with incidental findings of a 16p11.2 BP4-5 CNV consecutive to their participation in a biobank, reported that overall, they felt empowered and positively valued the diagnosis.^65^^,^^327^^,^^328^ In this review, we emphasize how integrating results from diverse data sources in terms of ascertainment, demographics, and ancestry, as well as analytical approaches and experimental settings, can help fill current knowledge gaps and deepen our understanding of the mechanisms underlying variability in expressivity and penetrance, with the hope that this will guide the development of personalized prevention and treatment strategies.

Deleterious enough to be enriched in clinical cohorts but not enough so to be absent from population cohorts, 16p11.2 BP4-5 is an ideal showcase example of pleiotropy, but we envision that the approaches described in this review can be adapted to better delineate and understand the pleiotropic spectrum of other recurrent CNVs and structural variants.

## Acknowledgments

This study was funded by the 10.13039/501100001711Swiss National Science Foundation (31003A_182632 to A.R.; 310030_189147 to Z.K.), Horizon2020 Twinning projects (ePerMed 692145 to A.R.), and the Department of Computational Biology (Z.K.) and the Center for Integrative Genomics (A.R.) from the 10.13039/501100006390University of Lausanne. It makes use of data generated by the DECIPHER community. Funding for the DECIPHER project was provided by the 10.13039/100010269Wellcome Trust (WT223718/Z/21/Z).

## Author contributions

C.A. and A.R. drafted the manuscript; C.A. created the figures; Z.K. made critical revisions; and all authors approved the final manuscript.

## Declaration of interests

The authors declare no competing interests.

## Web resources

ClinGen, https://search.clinicalgenome.org/kb/gene-dosage/region/ISCA-37400
DECIPHER, https://www.deciphergenomics.org/
Eagle, https://alkesgroup.broadinstitute.org/Eagle/downloads/tables/
GnomAD, https://search.clinicalgenome.org/kb/gene-dosage/region/ISCA-37400
IMPC, https://www.mousephenotype.org/
OMIM, https://www.omim.org/
USCS, https://genome.ucsc.edu/cgi-bin/hgLiftOver

## Supplemental information

Document S1. Table S1

Document S2. Article plus supplemental information
