# Supplementary material for: The pleiotropic spectrum of proximal 16p11.2 CNVs
Source: Am J Hum Genet. 2024 Sep 26;111(11):2309–46. doi: 10.1016/j.ajhg.2024.08.015 (PMC11568765; doi:10.1016/j.ajhg.2024.08.015)
Supplement: Document S1. Table S1 [file mmc1.pdf]

The American Journal of Human Genetics, Volume *111*

Supplemental information

The pleiotropic spectrum of proximal 16p11.2 CNVs

Chiara Auwerx, Zoltán Kutalik, and Alexandre Reymond

SUPPLEMENTAL TABLES

Gene	IMPC	Lethality	reproductive	growth/size	metabolism/adiposity	behavior/neurologic	cardiovascular	limbs/digits/tail	skeleton	immune/hematologic	muscle	pigmentation	craniofacial	ear/hearing	endocrine/exocrine	eye/vision
Aldoa	yes	preweaning (-/-)														
Asphd1	yes															
AI467606	yes	preweaning* (-/-)														
4930451I11Rik	yes															
Cdipt	yes	embryonic (-/-)														
Coro1a	yes															
Doc2a	no															
Gdpd3	yes															
Hirip3	yes															
Ino80e	yes															
Kctd13	yes															
Kif22	no															
Mapk3	no															
Maz	yes															
Mvp	yes															
Pagr1	no															
Ppp4c	yes															
Prrt2	yes															
Qprt	no															
Sez6l2	yes															
Spn	yes															
Taok2	yes															
Tbx6	no															
Tlcd3b	yes															
Tmem219	yes															
Ypel3	yes															
Zg16	yes															
Bola2^{+}	yes															
Slx1b^{+}	yes															
Sult1a1^{+}	yes	preweaning* (+/-)														
**TOTAL**	**24**	**4**	**3**	**9**	**9**	**9**	**5**	**4**	**4**	**6**	**1**	**2**	**1**	**3**	**2**	**5**

Table S1. International Mouse Phenotyping Consortium 16p11.2 BP4-5 mouse models.
Alphabetical list of mouse genes orthologous to human 16p11.2 BP4-5 genes and whether (yes) or not (no) a knockout was generated by the International Mouse Phenotyping Consortium (IMPC; accessed March 2024). Subsequent columns indicate different phenotypes that were assessed, with black, white, and grey cells indicating whether the system was significantly affected, not affected, or not assessed, respectively. All systems for which at least one model exhibited a phenotype are shown. The total number of affected models is indicated in the last row. Lethality is assessed through specific viability protocols and stages are indicated, along with whether observed in homozygous (-/-) or heterozygous (+/-) mice models; * indicates that lethality is not fully penetrant. Genes in the flanking breakpoint regions are at the bottom and marked with $^{+}$.
